# Placenta-Specific Genes, Their Regulation During Villous Trophoblast Differentiation and Dysregulation in Preterm Preeclampsia

**DOI:** 10.3390/ijms21020628

**Published:** 2020-01-17

**Authors:** Andras Szilagyi, Zsolt Gelencser, Roberto Romero, Yi Xu, Peter Kiraly, Amanda Demeter, Janos Palhalmi, Balazs A. Gyorffy, Kata Juhasz, Petronella Hupuczi, Katalin Adrienna Kekesi, Gudrun Meinhardt, Zoltan Papp, Sorin Draghici, Offer Erez, Adi Laurentiu Tarca, Martin Knöfler, Nandor Gabor Than

**Affiliations:** 1 Systems Biology of Reproduction Lendulet Group, Institute of Enzymology, Research Centre for Natural Sciences, H-1117 Budapest, Hungary; szilagyi.andras@ttk.hu (A.S.); gelzsolt@gmail.com (Z.G.); peter0kiraly@gmail.com (P.K.); ammdemeter@gmail.com (A.D.); palhalmi@gmail.com (J.P.); ho12ember@gmail.com (K.J.); 2 Perinatology Research Branch, Eunice Kennedy Shriver National Institute of Child Health and Human Development, National Institutes of Health, U.S. Department of Health and Human Services, Bethesda, MD 20692, and Detroit, MI 48201, USA; romeror@mail.nih.gov (R.R.); yxu@med.wayne.edu (Y.X.); erezof@bgu.ac.il (O.E.); atarca@med.wayne.edu (A.L.T.); 3 Department of Obstetrics and Gynecology, University of Michigan, Ann Arbor, MI 48109, USA; 4 Department of Epidemiology and Biostatistics, Michigan State University, East Lansing, MI 48824, USA; 5 Center for Molecular Medicine and Genetics, Wayne State University, Detroit, MI 48201, USA; 6 Detroit Medical Center, Detroit, MI 48201, USA; 7 Department of Obstetrics and Gynecology, Florida International University, Miami, FL 33199, USA; 8 Department of Obstetrics and Gynecology, Wayne State University School of Medicine, Detroit, MI 48201, USA; 9 Laboratory of Proteomics, Institute of Biology, Eotvos Lorand University, H-1117 Budapest, Hungary; gyorffy.balazs88@gmail.com (B.A.G.); kakekesi@ttk.elte.hu (K.A.K.); 10 Maternity Private Clinic of Obstetrics and Gynecology, H-1126 Budapest, Hungary; hupuczi.petronella@maternity.hu (P.H.); pzorvosihetilap@maternity.hu (Z.P.); 11 Department of Physiology and Neurobiology, Eotvos Lorand University, H-1117 Budapest, Hungary; 12 Department of Obstetrics and Gynecology, Reproductive Biology Unit, Medical University of Vienna, Vienna A-1090, Austria; gudrun.meinhardt@meduniwien.ac.at (G.M.); martin.knoefler@meduniwien.ac.at (M.K.); 13 Department of Obstetrics and Gynecology, Semmelweis University, H-1088 Budapest, Hungary; 14 Department of Computer Science, Wayne State University College of Engineering, Detroit, MI 48202, USA; sorin@wayne.edu; 15 Department of Obstetrics and Gynecology, Soroka University Medical Center, Ben-Gurion University of the Negev, Beer-Sheva 84101, Israel; 16 1st Department of Pathology and Experimental Cancer Research, Semmelweis University, H-1085 Budapest, Hungary

**Keywords:** development, immune tolerance, metabolism, microarray, omics, transcriptional network

## Abstract

The human placenta maintains pregnancy and supports the developing fetus by providing nutrition, gas-waste exchange, hormonal regulation, and an immunological barrier from the maternal immune system. The villous syncytiotrophoblast carries most of these functions and provides the interface between the maternal and fetal circulatory systems. The syncytiotrophoblast is generated by the biochemical and morphological differentiation of underlying cytotrophoblast progenitor cells. The dysfunction of the villous trophoblast development is implicated in placenta-mediated pregnancy complications. Herein, we describe gene modules and clusters involved in the dynamic differentiation of villous cytotrophoblasts into the syncytiotrophoblast. During this process, the immune defense functions are first established, followed by structural and metabolic changes, and then by peptide hormone synthesis. We describe key transcription regulatory molecules that regulate gene modules involved in placental functions. Based on transcriptomic evidence, we infer how villous trophoblast differentiation and functions are dysregulated in preterm preeclampsia, a life-threatening placenta-mediated obstetrical syndrome for the mother and fetus. In the conclusion, we uncover the blueprint for villous trophoblast development and its impairment in preterm preeclampsia, which may aid in the future development of non-invasive biomarkers for placental functions and early identification of women at risk for preterm preeclampsia as well as other placenta-mediated pregnancy complications.

## 1. Introduction

The human placenta maintains pregnancy and supports the developing fetus by providing nutrition, gas-waste exchange, hormonal regulation, and an immunological barrier from the maternal immune system [1]. Most of these functions are provided by one particular cell type, the syncytiotrophoblast, which lies at the maternal-fetal interface [1,2,3,4]. This multinucleated cell is derived by the biochemical and morphological differentiation and fusion of the villous cytotrophoblasts [2,3,5,6,7,8,9,10,11,12,13]. During the developmental process of cytotrophoblast differentiation and transformation into the syncytiotrophoblast, there is a parallel loss and gain of cellular functions, such as the production of a set of placenta-specific or placenta-enriched molecules [2,3,14,15,16,17,18]. These include peptide and steroid hormones, structural and immune proteins, enzymes, and transcription factors. [14,15,16,17,19] Remarkably, separate observations have noted that many predominantly placenta-expressed (PPE) molecules are encoded by primate-specific gene clusters (e.g., chorionic gonadotropins (CGBs), galectins, microRNAs) [15,16,17,19,20,21,22,23,24,25,26], suggesting that the evolution of these clusters and their trophoblastic expression might have strongly impacted the complex regulation of pregnancy in humans.

An earlier microarray study on in vitro differentiating cytotrophoblasts identified expression changes of genes and gene clusters related to structural and functional differentiation of cytotrophoblasts [5,6]. Functional experiments utilizing cytotrophoblasts isolated from healthy term placentas found that the reprogramming of the villous trophoblast transcriptome during differentiation and the unique transcriptomic activity of the syncytiotrophoblast [27] were partly governed by cAMP through the protein kinase A (PKA) or Epac pathways [2,5,6,7,8,9,11,12,28,29,30,31,32]. To some extent, the morphological (syncytialization) and biochemical differentiation of cytotrophoblasts may be separate [11,12]: Epac signaling may regulate syncytialization while both Epac and PKA signaling may be involved in biochemical differentiation [2,31,33]. It was also revealed that the onset and/or progression of cytotrophoblast differentiation was controlled by EGF signaling [34,35,36,37] as well as ERK1/2 [37,38], p38 [39,40], and SFK [41,42] kinase pathways. Among the transcription factors involved in this process, ESRRG [19,43,44], GATA2/3 [19,45,46,47], GCM1 [19,48,49], TEF5 [19,50,51], and TFAP2A/C [8,9,18,19,52,53,54,55,56] have been most thoroughly described.

Preeclampsia, a life-threatening obstetrical syndrome related to placental dysfunction [32,57,58,59,60,61,62,63,64,65], is associated with dysregulated expression or function of many PPE proteins (e.g., CGB, LEP), transcription factors (e.g., GCM1, ESRRG), and signaling and kinase pathways (e.g., ERK1/2, p38), especially when it develops preterm [19,57,66,67,68,69,70,71,72,73,74,75]. Based on the observations that the volume [76] and cellular architecture [67,68,69,77,78,79] of villous trophoblasts are severely impacted in preterm preeclampsia, it has been proposed that either the entire cytotrophoblast differentiation program [80], or particularly syncytialization [81,82,83,84,85,86,87], are affected in preterm preeclampsia, although neither have been proven at the systems level yet. Thus, it would be imperative to explore the dynamic transcriptomic changes during normal cytotrophoblast differentiation to understand its dysregulation in preterm preeclampsia.

Our group recently uncovered characteristic placental transcriptomic changes and identified distinct gene modules and their hub transcription factors that are involved in the development of maternal or fetal disease conditions associated with preterm preeclampsia [57]. We found that many of these hub transcription factors and their target genes were predominantly expressed by villous trophoblasts, supporting their central role in the development of preeclampsia. To address the important gaps in our understanding of normal and pathologic villous trophoblast developmental pathways, we applied in this current study a systems biology approach and in silico and in vitro analytic methods (Figure S1) that include the analysis of microarray datasets. We investigated the following areas: (1) the importance of the expression, biological function, and regulatory network of PPE genes in villous trophoblast functions and differentiation; (2) the grouping of genes differentially expressed during villous trophoblast differentiation into regulatory modules and clusters, their enrichment in biological processes and cell compartments, and their common regulatory pathways; and (3) the association of villous trophoblastic gene modules and their hub transcription factors with the placental transcriptomic changes in preterm preeclampsia.

## 2. Results

### 2.1. Identification of Predominantly Placenta-Expressed Genes

**Figure 1:**
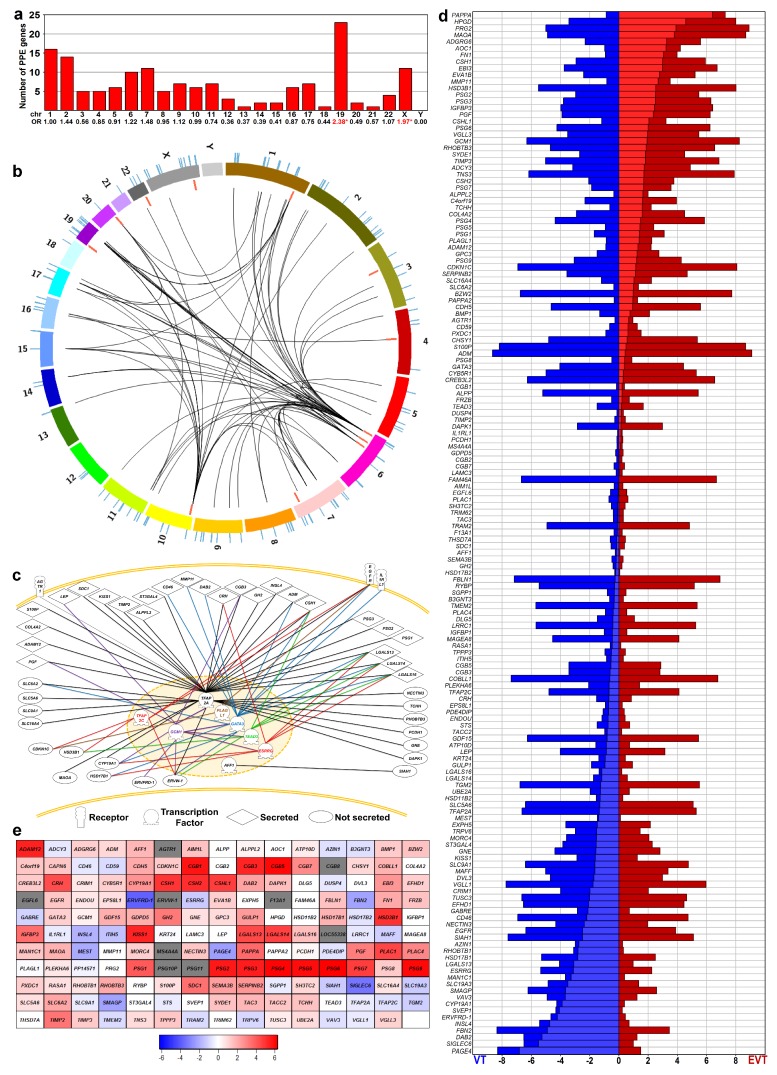
Predominantly placenta-expressed genes (PPE). (**a**) Human predominantly placenta-expressed (PPE genes identified by analyzing BioGPS microarray data. The bar chart displays the chromosomal distribution of PPE genes. Numbers denote the odds ratio (OR) of chromosomal PPE gene enrichment; stars depict significance. (**b**) Circos visualization of the genomic localization of PPE genes. Colored arcs indicate chromosomes; lines in the outer and inner circles show the localization of PPE genes. Red lines depict PPE transcription regulatory (TR) genes, while blue lines indicate PPE target genes. Curved black lines demonstrate the functional connections between seven TR genes and 42 target genes. The enrichment of Chr6 for PPE TR genes (OR = 8.229, *p* = 0.029) and Chr19 for PPE target genes (OR = 2.324, *p* = 0.003) is clearly visible. (**c**) Functional connection between PPE genes. Network connections of various TR genes with target genes were depicted with different line colors using Pathway Studio. (**d**) Expression of PPE genes in villous (VT) and extravillous (EVT) trophoblasts. Horizontal bars show the absolute expression (compared to background signal) based on microarray data from Apps et al. [94]. Red represents EVT while blue depicts VT gene expression; lighter red and blue indicate the difference between the absolute expression values of EVT and VT. The vertical ordering of genes was based on the EVT-VT expression difference. (**e**) PPE gene expression during VT differentiation. Microarray data was obtained from primary VTs isolated from third trimester normal placentas (*n* = 3) and differentiated during a seven-day period. The largest differences in gene expression compared to Day 0 were visualized on the heat map. The color code depicts log_2_-fold change values; blue: down-regulated, red: up-regulated, grey: no available data.

Earlier, we showed the enrichment of PPE genes among differentially expressed genes in the placenta in preterm preeclampsia [57,88]. Herein, we investigated how these PPE genes are involved in villous trophoblast functions and differentiation, and whether their villous trophoblastic dysregulation may have pathologic significance in preterm preeclampsia. To identify PPE genes, we first mined BioGPS microarray and published data [15,17,57,89]. Among the 164 genes that met our stringent criteria (Table S1), 13 genes encode transcription regulatory proteins while 151 encode their potential targets. Interestingly, PPE genes are not randomly located in the genome, partly because many are members of gene families clustered in nearby loci. For example, Chr19 had the largest number of PPE genes and their highest enrichment (odds ratio [OR] = 2.38, *p* = 10^−4^), followed by Chr X (OR = 1.97, *p* = 0.025) (Figure 1a).

DAVID annotation of PPE genes confirmed their enrichment only in the placenta and revealed that their top enrichments in biological processes (e.g., ‘female pregnancy’, ‘reproductive process’, ‘tissue development’), molecular functions (e.g., ‘receptor binding’, ‘hormone activity’), KEGG pathways (e.g., ‘steroid hormone biosynthesis’), and UniProt keywords (e.g., ‘secreted’, ‘signal’, ‘glycoprotein’, ‘hormone’) were strongly associated with trophoblastic functions (Table S2).

### 2.2. Predominantly Placenta-Expressed Genes are Key to Villous Trophoblastic Functions

Although published functional data were unavailable or insubstantial for most of these PPE genes, a functional network of 54 genes could be constructed with Pathway Studio based on pathway, cell process, and molecular interaction data located in PubMed. Figure 1b depicts the chromosomal location of PPE genes encoding transcriptional regulators, their target proteins and their known network connections derived from published data [19,55,90], which are also shown in detail in Figure 1c. Within this network, the epidermal growth factor (EGF) receptor may transmit key extracellular signals to the trophoblast, as also evidenced in earlier investigations [34,35,36,37,91]. Signal transduction events in the trophoblast led to gene expression regulation by transcription regulatory proteins, from which TFAP2 family members have been well studied. TFAP2A, via the cAMP-PKA signaling cascade, is central to trophoblastic functions, while TFAP2C is part of the trophectoderm core transcriptional regulatory circuitry [8,9,18,19,52,53,54,92]. GCM1 is key in trophoblastic fusion and differentiation [19,48,49,93], and GATA factors [19,45,46,47] and TEF5 [19,50,51] drive trophoblast-specific gene expression, while ESRRG is pivotal for trophoblastic metabolic functions [19,43,44]. These transcription regulatory proteins are crucial in regulating the expression of several PPE genes encoding secreted trophoblastic proteins (e.g., hCG, hCS, Chr19 galectins, CYP19) [8,9,19,44,48,54].

### 2.3. Predominantly Placenta-Expressed Genes Are Primarily Expressed in Villous Trophoblasts

Subsequently, we explored the growing body of functional evidence that indicates PPE genes may be chiefly expressed by the trophoblast, by studying the immunostaining data available in the Human Protein Atlas and previous reports cited in the Introduction on 151 PPE proteins. Of them, 96% (145/151) had evidence for trophoblastic expression, 37% were expressed in the villous mesenchyme, 56% in the villous endothelium, and 44% in decidual stromal cells. Of importance, the immunostaining intensity for 78% (118/151) of PPE proteins was the strongest in villous trophoblasts, while 48% (72/151) of these proteins’ expression was unique to villous trophoblasts.

To validate these findings at the level of transcripts, we re-analyzed three microarray datasets by comparing gene expressions of villous trophoblasts and extravillous trophoblasts [94,95,96]. Of the genes encoding 164 PPE proteins, expression data were found for 143 (87%) in all datasets. Given that gene expression patterns for both extravillous and villous trophoblasts were similar in all studies (Figure S2) despite their methodological differences, we used one dataset [94] for further analysis. In accordance with our immunostaining data, more genes had higher expression in villous trophoblasts over extravillous trophoblasts (*n* = 45) than in extravillous trophoblasts over villous trophoblasts (*n* = 28), while 45 genes had the same expression level in both cell lineages (Table S3, Figure 1d).

### 2.4. Most Predominantly Placenta-Expressed Genes are Differentially Expressed During Villous Trophoblast Differentiation

**Figure 2:**
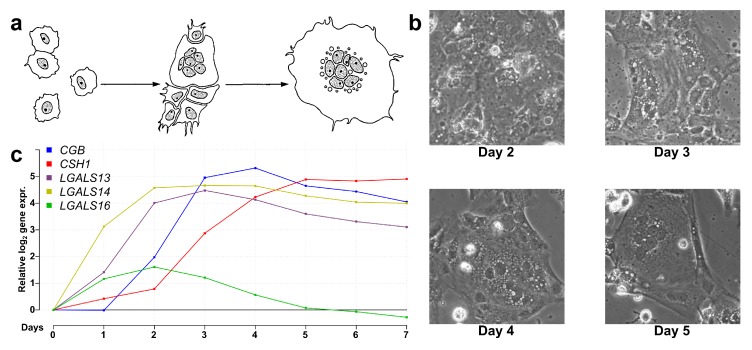
Microarray validation with qRT-PCR. (**a**) Schematic visualization of villous trophoblast differentiation adapted with permission from Hubert et al. [97]. (**b**) Brightfield images (250×) of differentiating and fusing villous trophoblasts on days 2, 3, 4, and 5 of differentiation. (**c**) Line graph showing the relative gene expression change of five genes with predominant placental expression (PPE) from day 0 to day 7 during trophoblast differentiation.

Next, we aimed to explore how PPE genes are regulated in the context of the transcriptomic and functional changes during villous trophoblast differentiation. Therefore, we performed a 7-day RNA microarray and qRT-PCR expression profiling experiment. As illustrated in Figure 2, the classical morphological changes of villous trophoblast differentiation were detected (Figure 2b), and the expression changes of five PPE genes were in accordance with previously published data [19,57] (Figure 2c). There was a good correlation between microarray and validation qRT-PCR data throughout the differentiation process for all eight validated genes, thus further corroborating our experiment (Figure S3). The correlation between RNA and protein levels was demonstrated for *LGALS13* and *LGALS14* in earlier work [19].

Among the 155 PPE genes with a valid probe on the array, 32 genes with high (≥4 fold above background) expression from day 0 to day 7 were observed, suggesting that these have important functions in villous trophoblasts regardless of the differentiation state. In addition, 91 PPE genes displayed differential expression (highest fold change of ≥2 compared to day 0) during the seven-day interval. Most of these genes (*n* = 76) showed increasing expression, underlining the remarkable gain of function during villous trophoblast differentiation (Figure 1e). In accordance with the immunostaining data, we found that 78% (121/155) of PPE genes were involved in either the steady-state or development-related functions of villous trophoblasts. All these findings indicate that most PPE genes are important for villous trophoblast functions, and their impaired expression in preterm preeclampsia may be related to the dysregulation of these genes during villous trophoblast development. Substantiating this finding, we found that among differentially expressed genes in the placenta in preterm preeclampsia [57], genes predominantly expressed by villous trophoblasts were more abundant (61%).

### 2.5. Microarray Analysis Reveals Global Transcriptomic and Functional Changes During Villous Trophoblast Differentiation

Subsequently, we analyzed the total transcriptomic changes during normal villous trophoblast differentiation with our microarray data. Modern RNA microarray technology enabled us to perform differential expression analysis on a much larger set of genes and to obtain higher temporal resolution than in earlier studies [5,6]. To obtain an overall picture of the differentiation processes, we calculated the fold changes of all genes at all time-points compared to day 0 (Figure S4). We identified 1937 differentially expressed genes, including 908 up-regulated and 1029 down-regulated genes (Table S4), which suggested a large functional reorganization of villous trophoblasts during the transition from cytotrophoblasts to the syncytiotrophoblast.

**Figure 3:**
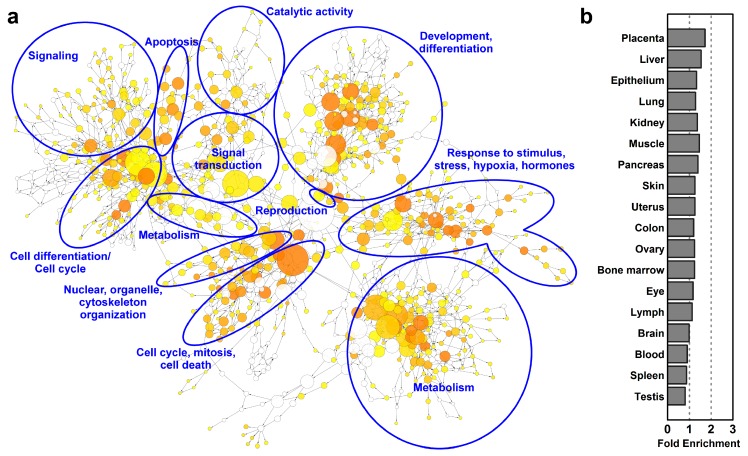
Gene-regulatory networks, biological processes and tissue enrichments in differentiating villous trophoblasts. (**a**) The network of biological processes enriched among differentially expressed (DE) genes was created by BiNGO and visualized with Cytoscape. Sizes of the circles relate to the number of genes involved in the biological processes and colors refer to *p*-values. The groups of most enriched biological processes were manually circled and labeled. The color code depicts *p*-values. (**b**) The UniProt tissue enrichments among DE genes were assessed with DAVID and are represented in a bar chart in the order of their enrichment significance value.

To obtain a global view on functional changes during villous trophoblast differentiation, we analyzed the biological processes enriched among differentially expressed genes by DAVID (Table S4) and visualized their network by using the BiNGO [98] plugin in Cytoscape. As a good confirmation of our analysis, many of the most enriched biological processes (Figure 3a) have been delineated by earlier studies on villous trophoblast differentiation [5,6,99]. There is a wide range of enriched biological processes (*n* = 952) related to the morphological and functional transformation from a proliferative cytotrophoblast into a metabolically and hormonally active end-differentiated syncytiotrophoblast, including ‘programmed cell death’, ‘tissue development’, ‘apoptotic process’, and ‘cell cycle’ as top enriched GO biological processes among differentially expressed (DE) genes (Table S4).

These biological processes are regulated by various stimuli as indicated by the terms ‘response to stress’, ‘response to organic substance’, ‘cellular response to chemical stimulus’, ‘response to oxidative stress’, ‘response to oxygen-containing compound’, and ‘response to growth factor’ being among the top enriched GO biological processes among DE genes. The groups of most enriched biological processes were manually circled and labeled on Figure 3.

Of interest, the UniProt Tissue analysis by DAVID revealed that biological functions related to villous trophoblast differentiation were not only enriched in the placenta (1.7-fold), but also in other organs (1.2–1.5-fold) (Figure 3b). This finding reflected that the placenta is a multifunctional organ and that the villous trophoblast, beyond its specific functions, utilizes the molecular machinery of these organs for its metabolic, nutritive, exchange, and defense functions as well as the general machinery for cell cycle control.

### 2.6. Identification of Gene Modules and Module Groups From the Co-Expression Network During Villous Trophoblast Differentiation

**Figure 4:**
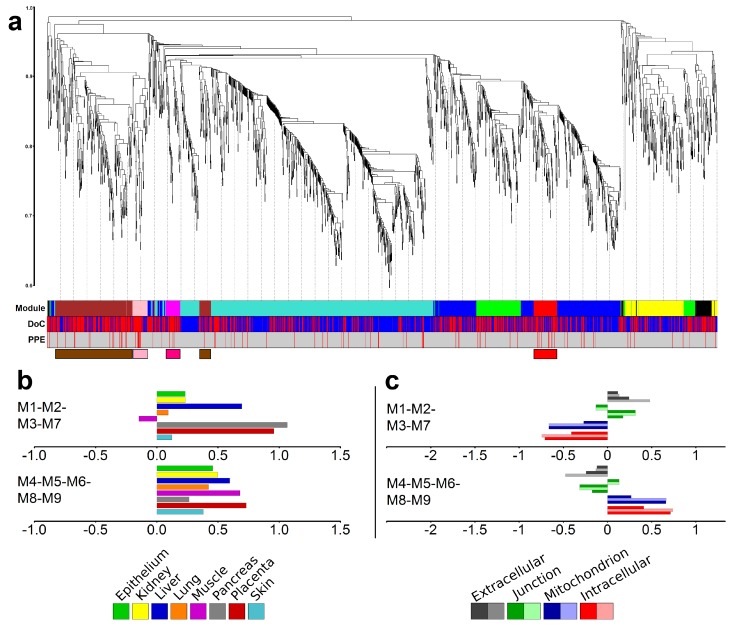
Modules of co-expressing genes. (**a**) Weighted co-expression network analysis (WGCNA) of differentially expressed (DE) genes identified five major and four minor modules. The y-axis represents the distance metric (1-TOM); the three levels of colored bars below the chart indicate the identified modules, the direction of change of expression (DoC; red: up-regulated, blue: down-regulated), and predominantly placental expressed (PPE) genes. The four modules enriched in PPE genes are depicted in the bottom with module-colored boxes. (**b**) Tissue enrichments of the combination of placental (M1-M2-M3-M7) and non-placental (M4-M5-M6-M8-M9) modules as analyzed with the DAVID bioinformatics tool. (**c**) Cellular component enrichments for the same two module groups as analyzed by DAVID.

Subsequently, Weighted Gene Co-expression Network Analysis (WGCNA) [100,101] was used on differentially expressed genes to define modules of genes with correlated expression. The procedure identified nine different co-expressing gene modules (M1–M9), indicated with different colors (Figure 4a, Table 1). Four of these modules (M1-brown, M2-pink, M3-magenta, M7-red) were found to be enriched in PPE genes. Thus, we distinguished between placental (M1, M2, M3, M7) and non-placental (M4, M5, M6, M8, M9) module groups (Table S5). Of note, the M1-brown module was also enriched in genes related to preterm preeclampsia [57].

When analyzing the gene modules with the DAVID bioinformatics tool, we observed differences in the tissue and cellular component distribution between genes in the placental and non-placental module groups (Figure 4b,c and Figure S5). The group of placental modules was enriched with placental, liver, and pancreatic tissue functions, while the group of the other five modules had no outstanding enrichment for any tissues (Figure 4b). The group of placental modules was enriched with extracellular and junction components and devoid of intracellular and mitochondrial components, while the opposite was true for the group of the other five modules (Figure 4c).

### 2.7. Major Differences in Pathway Perturbation of Placental and Non-Placental Modules

Next, we investigated placental and non-placental module genes regarding gene and pathway perturbations by utilizing the iPathwayGuide tool, which considers both the genes’ measured fold change and the accumulated perturbation propagated from any upstream genes. The perturbed pathways for each gene module are listed in Table S6. We observed large differences in the nature of the perturbed pathways by the expression changes in the placental-module and non-placental-module genes.

**Figure 5:**
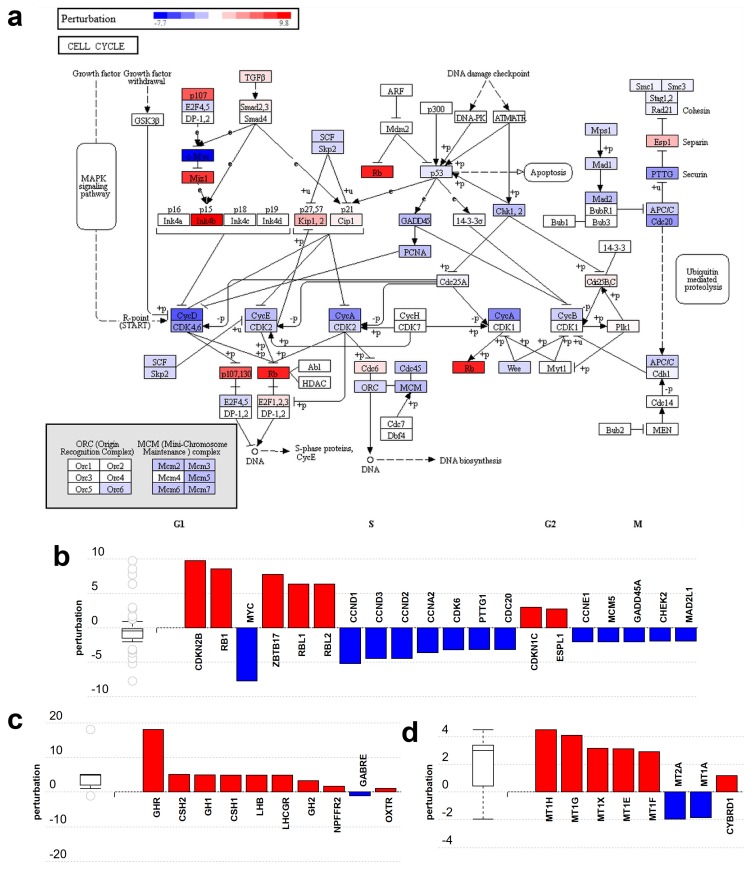
Gene perturbation analysis by iPathwayGuide: Perturbation of non-placental-module genes in the cell cycle pathway (KEGG:04110). (**a**) The pathway diagram is overlaid with the computed perturbation of each gene. The perturbation accounts both for the gene’s measured fold change and for the accumulated perturbation from any upstream genes. The highest negative perturbation (−7.7) is shown in dark blue, while dark red represents the highest positive perturbation (9.8). Note: For legibility, one gene may be represented in multiple places in the diagram and one box may represent multiple genes in the same gene family. For each gene family, the color corresponding to the gene with the highest absolute perturbation is displayed. (**b**) Gene perturbation bar plot. All the genes in the cell cycle pathway are ranked based on their absolute perturbation values. For each gene, the signed perturbation is represented with negative values in blue and positive values in red. The box and whisker plot on the left summarizes the distribution of all gene perturbations in this pathway. (**c**,**d**) show placental-module genes perturbed in the neuroactive ligand-receptor interaction pathway (KEGG:04080) and the mineral absorption pathway (KEGG:04978), respectively. Figures were generated by iPathwayGuide.

Among the 25 pathways significantly perturbed by non-placental-module gene expression changes, 10 were linked to the cell cycle, proliferation, carcinogenesis, and related signaling processes. Figure 5a depicts the perturbation of genes in the ‘cell cycle’ pathway (KEGG 04110). Genes that encode proteins promoting cell cycle progression, e.g., c-Myc *(MYC)* and cyclins (*CCN* genes), are down-regulated while tumor suppressors such as Rb *(RB1)* and p15 *(CDKN2B)* are up-regulated (Figure 5b).

Among the pathways perturbed by placental-module gene expression changes, two are significantly impacted: the ‘neuroactive ligand-receptor interaction’ pathway (Figure 5c) (KEGG:04080) and the ‘mineral absorption’ pathway (Figure 5d) (KEGG:04978). Both pathways are activated as most genes are up-regulated. Other less significantly affected pathways include ‘ovarian steroidogenesis’ as well as several metabolic and signaling pathways also involved in placental functions. These changes reflect the fact that the placenta functions as various organs and organ systems such as the kidney, gastrointestinal system, lungs, neurohormonal system, etc.

### 2.8. Major Differences in the Time Course of Gene Expression in Placental and Non-Placental Modules

**Figure 6:**
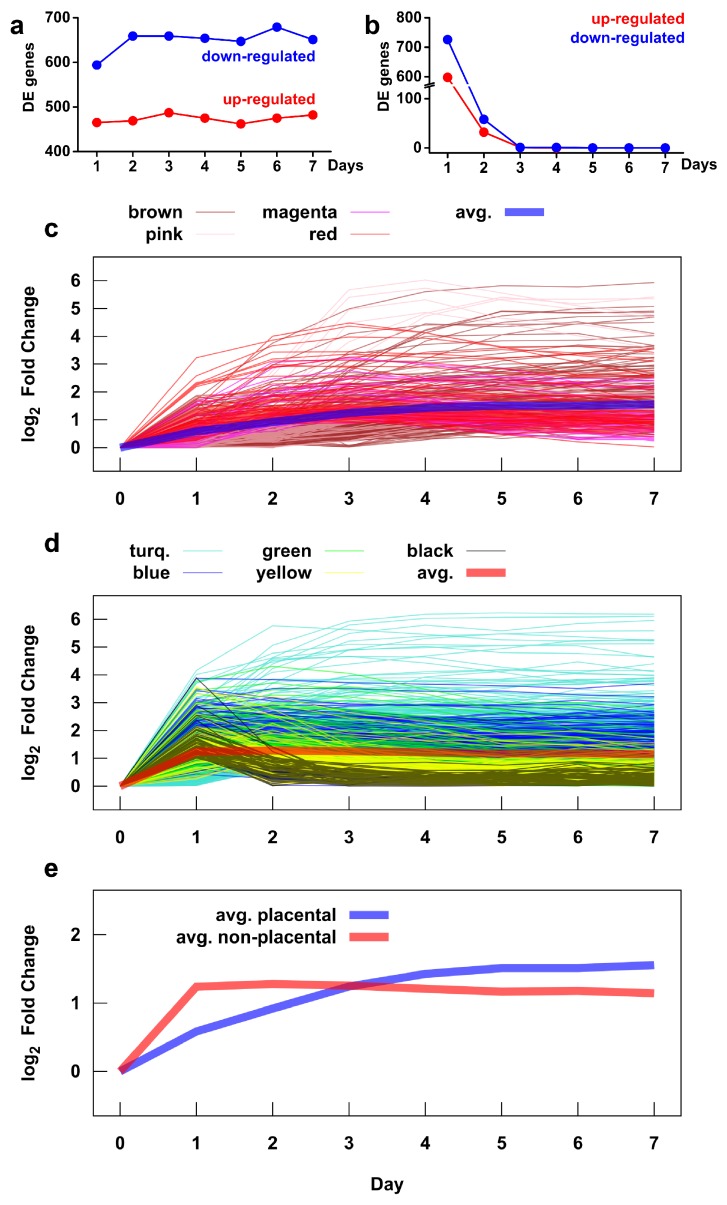
Time course of gene expression during villous trophoblast differentiation. The number of up- and down-regulated genes during differentiation was assessed by comparing mean gene expressions on a given day versus day 0 (**a**) and versus the previous day (**b**). The time course of expression changes, measured as the absolute value of the log_2_ fold change, of genes in placental (**c**) and non-placental (**d**) gene modules. The colors correspond to the module colors defined in Figure 4. The average expression time courses for the two module groups are shown in (**c**,**d**) with thick lines and are compared directly in (**e**). See Figure S6 for expression time courses for each gene module.

We also investigated the time course of transcriptomic changes to infer the dynamics of functional alterations (Figure 6 and Figure S6). The most important findings include the following: (1) 55% (1059/1937) of differentially expressed genes already showed differential expression on the first day of differentiation; (2) there were no genes with differential expression between subsequent days after the third day; and (3) a larger number of genes were down-regulated than up-regulated throughout the seven-day test (Figure 6a,b). Of interest, the time courses of gene expression in the placental and non-placental module groups were markedly different, with most genes in the non-placental group showing a large change by the first day, while genes in the placental group had a slower rate of expression change, extending into the latter days (Figure 6c–e). This finding is supported by earlier studies showing similar pattern of expression change for several placental (*CGB* genes, *CSH* genes) and non-placental (e.g., *TIMP3*) module member genes. Figure S6 shows this general pattern even though the time courses of gene expression in individual modules widely differ within the module groups.

### 2.9. Identification of Genes with High Expression Change

Given the small number of genes with changing expression after the first day, a classical time-course analysis of biological process enrichments could not be applied. Instead, we looked at the biological functions of clusters of genes that followed distinct time courses of expression change, similarly reported in a previous study [5]. However, we utilized a different clustering method on a different set of genes. Since PPE genes had a higher mean differential expression than all differentially expressed genes (6.2-fold vs. 3.1-fold, *p* = 3.5 × 10^−31^), we hypothesized that genes with the highest expression change are the most relevant for villous trophoblast functions, and thus restricted our analysis to this subset. We identified these genes by analyzing the enrichment of PPE genes among differentially expressed genes as a function of log_2_-fold change thresholds (Figure S7). Given that the slope of the ORs for PPE genes increased remarkably, starting at the 2.5 log_2_-fold (~5.7-fold) change, we chose this threshold. This threshold set the top 1.4% of all genes (*n* = 204, Table S7), which had 22.9-fold enrichment for PPE genes. These high expression-change (HEC) genes were over-represented on Chr19 (OR = 1.91, *p* = 0.0028) (Figure S8A). The subset of PPE genes among HEC genes were over-represented on Chr19 (OR = 12.17, *p* < 0.0001) and Chr17 (OR = 2.76, *p* = 0.0368) as a result of the large gene clusters located on these chromosomes (Figure S8B).

### 2.10. High Expression Change Gene Clusters Point to Temporal Changes in Structural and Functional Rearrangements of Differentiating Villous Trophoblasts

**Figure 7:**
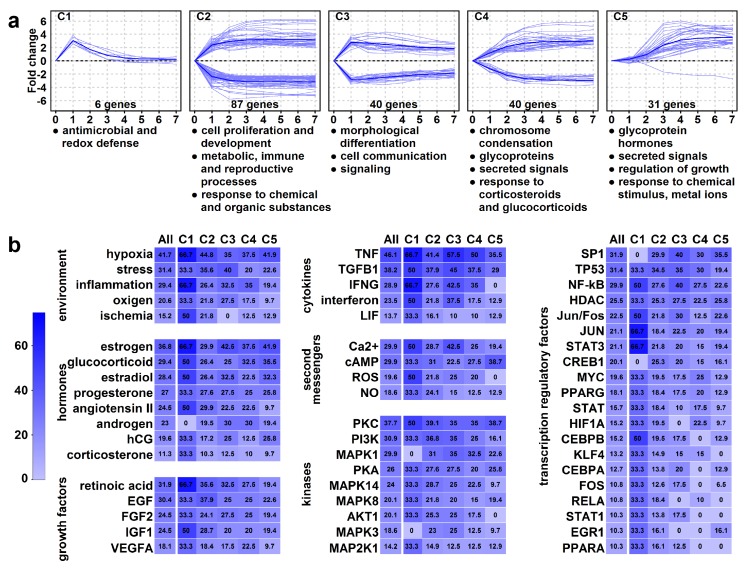
Genes with high expression change (HEC). (**a**) The graphs show the temporal expression pattern of genes in the clusters. Individual gene expression data (log_2_-fold change compared to day 0 is depicted with light blue lines, while mean cluster gene expression data is shown with dark blue lines. Cluster enrichments were assessed using DAVID, the significance threshold was set at FDR < 0.2. The biologically most relevant, terms were listed below the clusters. (**b**) The common regulators of clusters were analyzed using Pathway Studio 9.0, the significance threshold was set at *p* < 0.05. The percentage of HEC genes (“All”) or genes in clusters (C1–C5) regulated by various environmental stimuli or physiological regulators are depicted in heat maps. The color code depicts percentages.

Next, we performed a hierarchical cluster analysis of these 204 HEC genes, revealing five clusters (C1–C5) (Figure 7a). The analysis of genes and enrichments (Table S7) in these clusters pointed to the temporal changes leading to structural and functional villous trophoblast rearrangements:

An immediate and transient gene expression change characterized *C1 cluster* (*n* = 6) genes. These genes had immediate up-regulation and subsequent down-regulation and contained a peroxisome component and antimicrobial peptide genes, suggesting that an early defense against oxidative stress and infections is a key prerequisite for the syncytiotrophoblast that is in direct contact with maternal blood.

An immediate and persistent gene expression change characterized *C2* and *C3 clusters*. *C2 cluster* (*n* = 87) genes had immediate up- or down-regulation and constant expression from day 3. Down-regulated genes included cell cycle controllers (e.g., cyclins) and transcription factors *(ID2, AP1 factors, KLFs*), which may keep villous trophoblasts in proliferation [102,103,104]. Up-regulated genes are involved in development, steroid synthesis (*CYP19A1*, *HSD3B1*), metabolic, immune tolerance (*LGALS14*, *PSGs*), and reproductive processes [2,5,6,9,14,15,16,17,18,19,20,105], pointing to functional differentiation. *C3 cluster* genes (*n* = 40) had immediate up- or down-regulation with peak on day 1. Down-regulated genes included cyclins and cell adhesion molecules, while up-regulated genes comprised *CRH*, steroidogenic enzymes, and keratins, pointing to morphological differentiation and cell communication/signaling.

A slower and persistent gene expression change characterized *C4* and *C5 clusters*. *C4 cluster* (*n* = 40) had a slower change in expression with cell division genes down-regulated and the *PSGs*, *LGALS13*, and transport genes up-regulated. The enrichments with chromosome condensation, glycoproteins, secreted signals, and placental functions suggest that villous trophoblasts reach almost a full morphological and functional differentiated state when switching on/off genes in this cluster. *C5 cluster* (*n* = 31) genes, except for one, up-regulated in a delayed pattern, characteristic of mature syncytiotrophoblast functions inclusive of hormonal regulation (*CSHs*, *CGBs*, *GHs*) of pregnancy, metabolism, and growth as well as protection against oxidative stress and metal toxicity (metallothioneins) [2,5,6,9,14,15,16,17,18,19,20,106,107].

### 2.11. Identification of Upstream Regulators of High Expression Change Gene Clusters

The analysis of common regulators of clusters with immediate expression change (*clusters 1–3*) revealed cytokines (e.g., TNF, TGFB1), hormones (estrogen, glucocorticoids, progesterone, angiotensin), growth factors (e.g., retinoic acid, EGF, FGF, IGF), and environmental factors (e.g., oxygen, stress, inflammation), which critically regulate events early in differentiation. Their signals are transmitted predominantly by various second messengers (e.g., calcium, cAMP, ROS), kinases (e.g., PKC, PI3K, MAPK), and transcription factors (e.g., SP1, TP53, NF-kB, AP1) (Table S8, Figure 7b).

Later events (*clusters 4–5)* are still substantially impacted by cytokines, hypoxia, and inflammation. However, the effect of growth factors is less pronounced while the effects of estrogen, progesterone, and human chorionic gonadotropin are more prominent. Among the most potent intracellular regulators of late signaling events are cAMP, PKC, PKA, and SP1. Strikingly, the effect of cAMP is the greatest on the *C5 cluster*, suggesting it fulfills a major role later in differentiation and in the regulation of placenta-specific functions.

Many of the upstream regulatory factors were described by earlier studies [5,6,7,8,9] focusing on regulators of villous trophoblast differentiation; our results corroborate their findings. We also must take caution given that some of the observed gene expression changes may have been barely due to the influence of stress and recovery of villous trophoblasts after isolation and seeding; therefore, some of the predicted upstream regulators only induce changes in vitro but are not involved in in vivo regulation. Nevertheless, our data indicate that a complex network of signaling molecules within the uterine microenvironment are involved in the induction and regulation of villous trophoblast differentiation, and their altered expression or function may be involved in the development of preterm preeclampsia.

### 2.12. Identification of the Transcription Regulatory Network During Villous Trophoblast Differentiation

Next, we also aimed to identify upstream regulatory factors from our villous trophoblast differentiation microarray data that may regulate DE genes during differentiation. Therefore, we defined a set of 1802 transcription regulatory (TR) genes as described in the Supplementary Information. Of these 1802 TR genes, 220 were differentially expressed. Correlation coefficients were calculated from the microarray gene expression data (eight days for each gene) between each differentially expressed TR gene and all differentially expressed genes. A co-expression network was determined by connecting gene pairs with an absolute correlation coefficient >0.9 to indirectly infer regulatory connections between TRs and their target genes.

**Figure 8:**
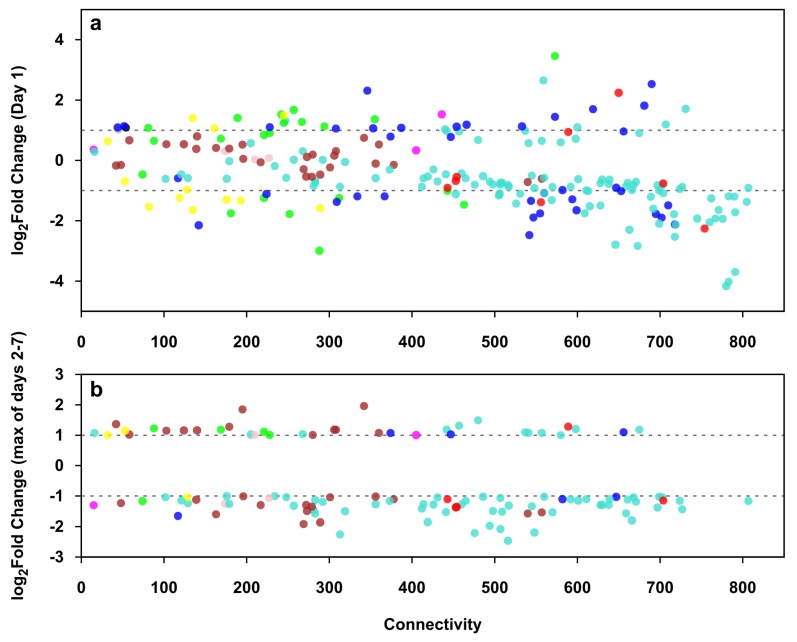
The expression and connectivity of differentially expressed transcription regulatory genes during villous trophoblast differentiation. Scatter plots show the relation of connectivity and expression change of transcription regulatory (TR) genes on day 1 (**a**) and on days 2 to 7 (**b**) of trophoblast differentiation. A co-expression network was calculated from the co-expression matrix of 220 differentially expressed TR genes and all 1937 differentially expressed genes, keeping only connections with a Pearson correlation coefficient *r* ≥ 0.9. Connectivity for each TR gene was defined as the number of its neighbors in the co-expression network. Fold changes in the expression of TR genes were calculated between each day and day 0 of the seven-day experimental differentiation period. Differentially expressed TR genes (|FC| ≥ 1 on any day) are shown in their respective module colors. (**b**) only shows TR genes not differentially expressed on Day 1.

Figure 8 shows the relation of connectivity (degree of the TR gene in the co-expression network) and expression change of differentially expressed TR genes of trophoblast differentiation on day 1 (Figure 8a) and on days 2 to 7 (Figure 8b), the latter showing only the TR genes not differentially expressed on day 1 (Figure 8b). On day 1, TR genes in the non-placental modules were already mostly differentially expressed, while on days 2 to 7, most of the placental module TR genes became differentially expressed. The majority of differentially expressed TR genes were down-regulated in both time periods. TR genes with higher connectivity tended to have a higher fold change, and those with the highest connectivity belong mostly to non-placental modules. Differentially expressed TR genes showed the same general dynamics as other DE genes during differentiation: a rapid and profound initial change in the regulation of non-placental gene modules, followed by slower and more limited placenta-specific transcriptional changes, leading to the completion of trophoblast differentiation.

### 2.13. Different Transcription Factors Regulate Placental and Non-Placental Modules

**Figure 9:**
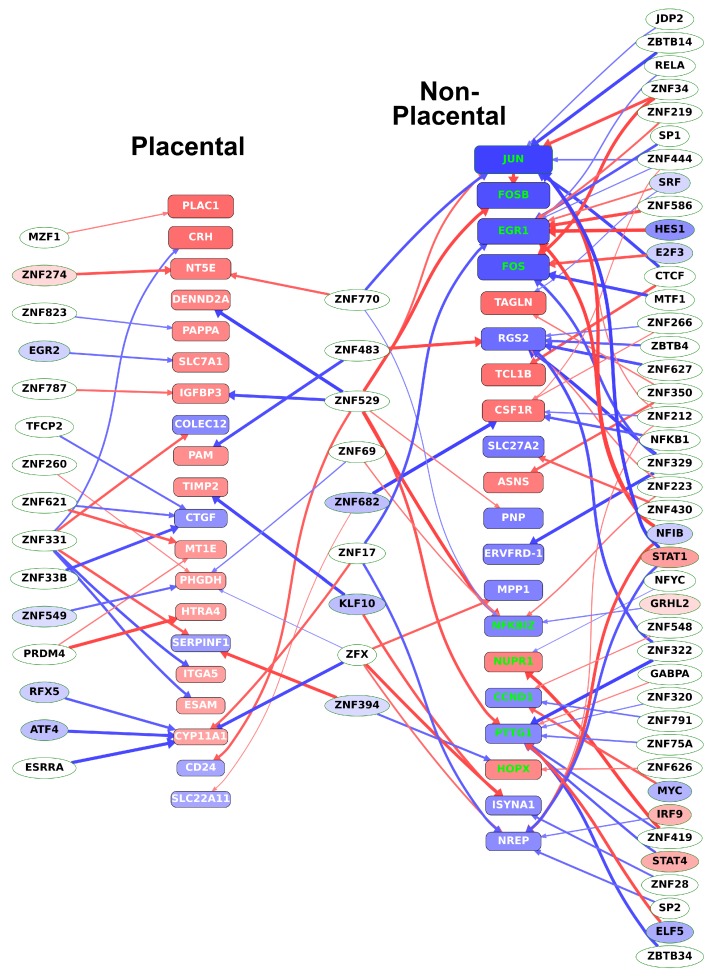
Transcription factors regulating key target genes in villous trophoblast differentiation. The transcription factors regulating the top 20 (defined by absolute fold change) DE genes in both the placental and the non-placental module groups were determined from DNaseI footprinting data as described in Materials and methods. Target genes (placental and non-placental) are represented by rounded boxes. Box size represents absolute log_2_ fold change and the box color represents log_2_ fold change with a color scale from blue through red indicating negative through positive values. Target genes that are transcription regulatory (TR) genes are highlighted in green. Transcription factors are represented by ellipses, where differentially expressed TRs are colored by log_2_ fold change as above. Arrows indicate binding of the transcription factor to a promoter region of the target gene but are only shown if the absolute gene expression correlation coefficient between them is > 0.6. Thicker arrows indicate higher correlation. Arrow color indicates the sign of the correlation coefficient (blue: negative; red: positive). (Figure created with Tabnetviz (git.io/tabnetviz)).

To also reveal direct evidence for TR—target gene connections, DNaseI footprinting datasets were used to identify transcription factors bound to the regulatory regions of DE genes, listed in Table S9. We found most upstream transcription factors to be uncharacterized zinc finger proteins. Most importantly, we identified three factors, *KLF10*, *ZNF394*, and *ZNF682*, which regulate at least 4% of both placental-module and non-placental-module genes (Table S9). Among these factors, *KLF10* is a transcriptional repressor that plays a role in the regulation of the circadian expression of genes and the cell cycle [108,109] while *ZNF394* and *ZNF682* are yet uncharacterized. Most of these transcription factors (displayed in the middle column in Figure 9) had expression correlation coefficients of opposite signs toward the placental-module and non-placental-module genes and, thus, appeared to be involved in the switch between the rapid, non-placental stage, and the slower, placenta-specific stage of villous trophoblast differentiation.

As shown in Figure 9, among the differentially expressed TR genes regulating the most differentially expressed genes, *NFIB* and *STAT1* in JAK/STAT signaling regulate at least 10% of the non-placental module genes. These TR genes are involved in RNA transcription, mediation of cellular responses to interferons, cytokines, and growth factors, and the regulation of trophoblast proliferation [110]. Among transcription factors that regulate at least 5% of genes among the non-placental-module genes, *IRF9* and *STAT4* are also involved in JAK/STAT signaling and the regulation of trophoblast differentiation [94], while *E2F3, ELF5, MYC*, and *SRF* regulate trophoblast stem cell renewal, cell cycle, and differentiation [111,112,113,114].

In accordance with the data in Table 1, Figure 9 also shows that most of the top 20 non-placental-module DE genes are down-regulated while most of the top 20 genes in the placental modules are up-regulated, pointing to the switch from a proliferative state to an end-differentiated state with pronounced placental gene expression.

In summary, these data show that formerly characterized regulators and pathways of trophoblast differentiation are modulated by several transcription factors that are completely uncharacterized and, thus, may be of interest for future research.

### 2.14. Identification of Transcription Regulatory Genes in the Trophoblast Differentiation Co-Expression Network that are Key to Preterm Preeclampsia Development

**Figure 10:**
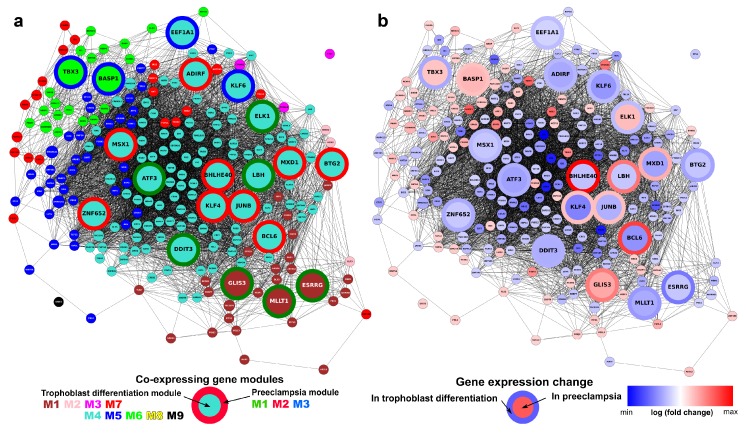
Co-expression network of transcription regulatory genes involved in villous trophoblast differentiation and preterm preeclampsia. The co-expression network of 220 DE transcription regulatory genes is shown; genes with an absolute expression correlation coefficient >0.9 are connected. The 20 genes that are also differentially expressed in preeclampsia [57] are shown in larger size. (**a**) The relationship of villous trophoblast (VT) differentiation gene modules and preterm preeclampsia DE gene modules. Node color represents VT differentiation gene module (colors are defined in Figure 4), while border color of the large nodes represents preeclampsia gene module as defined earlier [57]. (**b**) Relationship between fold changes during VT differentiation and in preterm preeclampsia. Node color indicates the log_2_ fold change of genes in VT differentiation, while border color of the large nodes indicates log_2_ fold change in preterm preeclampsia [57]. (Visualizations created with Tabnetviz (git.io/tabnetviz)).

Finally, we were interested in the identification of TR sub-networks involved in an altered villous trophoblast differentiation process that may be key to the development of preterm preeclampsia. Figure 10 shows the co-expression network of 220 differentially expressed TR genes in the villous trophoblast differentiation dataset. Highlighted among them are the 20 TR genes that are also differentially expressed in our preeclampsia placental microarray data [57] (Figure 10a). All the TR genes show good clustering by gene module, and the M4-turquoise module lies in the center of the network, where most preeclampsia-related TR genes are found. Comparing the fold changes of TR genes in villous trophoblast differentiation and preterm preeclampsia (Figure 10b), it is remarkable that a subset of seven genes (*BCL6, BHLHE40, ELK1, JUNB, KLF4, LBH, MXD1*) that change in opposite directions are tightly clustered. Six of the seven genes are down-regulated in villous trophoblast differentiation while up-regulated in preterm preeclampsia. All these TR genes belong to the M4-turquoise module in villous trophoblast differentiation, and five become members of the M2-red module involved in blood pressure elevation in preeclampsia [57], while two become members of the M1-green module associated with placental and fetal development problems in preeclampsia [57]. These data suggest that the expression changes of this subset of TR genes in the M4-turquoise module are central to preterm preeclampsia-related changes in trophoblast development and placental functions. Their altered expression and consequent downstream effects may lead to the perturbation of entire gene networks, the formation of trophoblastic disease gene modules, and the consequent placental/fetal developmental problems and increased trophoblastic release of blood pressure-elevating ‘toxins’ in preterm preeclampsia. Indeed, in vitro experiments in our preeclampsia study [57] indicated that, when hypoxic/ischemic conditions were present, the up-regulation of *BCL6* in the trophoblast induced the increased expression of genes in the M2-red blood pressure module, transcriptomic changes similar to that seen in preeclamptic placentas. This observation is of high importance, given that these seven TRs seem to be good drug-target candidates for the prevention of altered trophoblast development and malfunction in preterm preeclampsia.

## 3. Discussion

To address the important gaps in our understanding of villous trophoblast developmental pathways and their potential relation to placental disease pathways, especially to the development of preterm preeclampsia, we employed various in silico and in vitro methods and a systems biology approach. First, we analyzed available gene expression data from the literature and identified a set of 164 genes with predominant placental expression. In silico analysis of these PPE genes revealed that most were expressed in the end-differentiated syncytiotrophoblast and were strongly associated with trophoblastic functions. In addition, to detect yet unknown factors involved in villous trophoblast differentiation, we then performed a seven-day villous trophoblast differentiation experiment in vitro and determined daily changes in global gene expression by microarray analysis. Co-expression analysis of the 1937 DE genes revealed nine gene modules; among them, four were found to be enriched by PPE genes. A number of bioinformatics analyses revealed that the villous trophoblast differentiation process consists of (1) a rapid stage characterized by morphological changes and the down-regulation of cell proliferation-related, non-placental genes, and (2) a subsequent slower stage characterized by the up-regulation of genes related to placental functions. We indirectly identified key upstream signaling molecules of villous trophoblast differentiation by the analysis of online available pathway analysis databases. Then, we directly identified transcription factors that regulate villous trophoblast differentiation by the combined analysis of our microarray gene co-expression network and online available DNAseI footprinting data. Two sets of TRs regulating placental or non-placental modules were discovered, which are involved in the switching from the rapid non-placental to the subsequent slower placental stage of villous trophoblast differentiation. Importantly, within the co-expression network of differentially expressed TR genes, we identified a tight cluster of seven genes that showed opposite changes during villous trophoblast differentiation and preterm preeclampsia, suggesting that the altered expressions of these TR genes are at the center of preterm preeclampsia-related changes in trophoblast differentiation in placental development.

One limitation of this study was the moderate amount of cells of primary cells that could be isolated from the placentas in high quality. This allowed the determination of RNA levels but not protein levels. Another limitation is that RNA microarrays are unable to reveal the expression of polymorphic gene variants, which may have a role on preterm preeclampsia [115,116]. In future studies, next-generation sequencing may help reveal these variants.

In conclusion, by employing system biology tools, we uncovered the blueprint for villous trophoblast development and its impairment in preterm preeclampsia. These results may aid in future development of non-invasive maternal blood diagnostic or predictive biomarkers of placental functions and their use in the early identification of women at risk for placenta-mediated pregnancy complications. In addition, the identification of TRs in the center of trophoblastic disease gene networks in preterm preeclampsia may enable the development of novel drugs modulating their expression and thus targeting trophoblastic maldevelopment and dysfunction. The potential benefits of such a prospective therapeutic approach may be elucidated by future studies.

## 4. Materials and Methods

This is a summary of the experimental and bioinformatics methods used in the current study; a detailed description of these methods is available for the interested reader in the Supplementary Information. A flowchart of the procedures is provided in Figure S1, and a Venn diagram of the various gene sets we refer to is shown in Figure S9.

### 4.1. Experimental Procedures

#### 4.1.1. Collection and Culturing of Cytotrophoblasts

Placentas (*n* = 6) were collected from women who were enrolled at Hutzel Women’s Hospital of the Detroit Medical Center (Detroit, MI, USA). All participants provided written informed consent prior to the collection of samples for research purposes, according to protocols approved by the Institutional Review Boards of Wayne State University (approval number: #110605MP4F dated 10 March 2006) and the *Eunice Kennedy Shriver* National Institute of Child Health and Human Development, National Institutes of Health, U.S. Department of Health and Human Services (approval number: OH97-CH-N067, dated 4 December 1997). Placentas were taken from women with a normal pregnancy who delivered at term (≥37 weeks) an appropriate-for-gestational-age neonate [117]. Cytotrophoblasts were isolated, as published previously [19,57], and differentiated for seven days on collagen-coated plates. Brightfield images of differentiating trophoblast cultures were taken on selected days with a CKX41 inverted microscope (Olympus Corp., Center Valley, PA, USA). Cell viability, differentiation, and syncytialization were evaluated morphologically and by gene expression profiling of *ERVWE1* and *CGB3* [5,10,19]. The trophoblast cell culture was harvested for total RNA every 24 h. According to RNA yield, we ran either only microarray analysis (*n* = 1), only qRT-PCR validation (*n* = 3), or both (*n* = 2).

#### 4.1.2. RNA Microarray Analysis

RNA samples were processed and used for microarray analysis with Illumina HumanHT-12v4 Expression BeadChips (Illumina Inc., San Diego, CA, USA). Microarray data analysis was performed using the LIMMA package [118].

#### 4.1.3. qRT-PCR Validation

qRT-PCR validation was performed for nine genes: three transcription factors involved in trophoblast differentiation, five differentially expressed target genes, and one housekeeping gene. The target genes were selected based on their placenta-specific or placenta-enriched expression, as revealed by earlier work [19]. Total RNA was reverse transcribed, and TaqMan assays (Table S10) were used for gene expression profiling on the Biomark high-throughput qRT-PCR system (Fluidigm, San Francisco, CA, USA).

### 4.2. Bioinformatics Analyses

#### 4.2.1. Identification and Analysis of Predominantly Placenta-Expressed Genes

Predominantly placenta-expressed genes (*n* = 164) were identified from the human BioGPS microarray data [89] and published data [17,88] as previously defined [57]. Among PPE genes, transcription regulatory gene products were identified in the Uniprot (www.uniprot.org) and Gene Ontology (www.geneontology.org) databases, and their enrichments were analyzed with the PANTHER Classification System [119] and the DAVID Bioinformatics Resource [120,121]. Network connections of PPE genes were retrieved from previously published sources of evidence [19,55,90] and by the Pathway Studio 9.0 software (Elsevier Inc., Amsterdam, The Netherlands). Chromosomal localizations were visualized by Circos [122], and chromosomal enrichments were calculated using the Fisher’s exact test. The Human Protein Atlas (www.proteinatlas.org) and the GeneCards (www.genecards.org) records were used to analyze the tissue localization and expression pattern of PPE genes in the human placenta. The expression data of these genes in extravillous trophoblast and villous trophoblast lineages were obtained from published microarray datasets [94,95,96] and visualized by heat maps and bar charts.

#### 4.2.2. Identification and Analysis of Differentially Expressed Genes During Villous Trophoblast Differentiation

From our RNA microarray data (see the above-noted “RNA microarray analysis” section), dynamic changes in gene expression during differentiation were analyzed by comparing the mean expressions on a given day versus day 0. Differentially expressed genes (*n* = 1937) were identified if they had a highest fold change of ≥2 and false discovery rate (FDR)-adjusted *p*-values of <0.1 on any day compared to day 0. The temporal changes in gene expression throughout the seven-day experiment were compared in two different ways. Mean gene expressions were compared either on a given day versus day 0 or on a given day versus the previous day. In both analyses, the sets of differentially expressed genes were defined for each of the seven days.

#### 4.2.3. Functional Annotation and Pathway Analysis

Differentially expressed genes were analyzed for their enrichments in various biological processes and tissues by using DAVID [120,121]. The significance was set at a Benjamini-Hochberg FDR-adjusted *p*-value of <0.2. The network of biological processes among the differentially expressed genes and villous trophoblast differentiation pathways was determined by BiNGO [98]. Pathway Studio 9.0 and iPathwayGuide [123,124,125] were used for pathway analysis. iPathwayGuide scores the pathways by means of the Impact Analysis method [124,126,127]. Impact analysis is based on two types of evidence: (i) the over-representation of differentially expressed genes in a given pathway and (ii) the perturbation of that pathway computed by propagating the measured expression changes across the pathway topology. These aspects are captured by two independent probability values, pORA and pAcc, that are then combined in a unique pathway-specific *p*-value. The underlying pathway topologies, comprised of genes and their directional interactions, are obtained from the KEGG database [128]. A perturbation factor is computed for each gene on the pathway.

#### 4.2.4. Co-Expression Network Analysis

Weighted gene co-expression network analysis (WGCNA) [100,101] identified distinct modules among differentially expressed genes; these were further tested for enrichment in PPE and up-regulated genes with Fisher’s exact test. The modules were divided into two groups based on whether they were enriched in PPE genes.

#### 4.2.5. Genes with High Expression Change

Genes with high expression change were defined using a log_2_-fold change threshold determined with an analysis of the enrichment of genes in PPE genes among genes with fold changes above various thresholds. Based on this procedure, a log_2_-fold change threshold of 2.5 was used to define the “High Expression Change” (HEC) gene set, which includes 204 genes, ~1.4% of all genes with valid data from the microarray. A hierarchical cluster analysis of these HEC genes was performed to identify gene expression change patterns during trophoblast differentiation.

#### 4.2.6. Determining Transcriptional Networks from DNase Footprinting Data

To determine transcriptional regulatory networks, we used genome-wide maps of in vivo DNaseI footprints to identify occupied binding sites of sequence-specific transcription factors, following the method of Neph et al. [129] Two DNaseI footprinting datasets were used: a dataset obtained from H1 human embryonic stem cells differentiated into trophoblast cells by BMP4 treatment (ENCODE accession ENCSR179CDH) and a dataset obtained from human amniotic epithelial cells (HAEpiC; GEO accession GSM646560). The latter dataset was included because of its higher sequencing depth and resultant better coverage.

### 4.3. Data Availability

Primary villous culture microarray data were deposited in the Gene Expression Omnibus (GEO) database with the detailed hybridization information in accord with the MIAME guidelines and assigned the accession number GSE130339. Additional data are shown in Supplementary Tables and Supplementary Figures.

## Figures and Tables

**Table 1:** Summary of the gene modules in villous trophoblast differentiation.

Module	Number of Genes			Odds Ratio		Up-Regulated%	Transcription Regulatory Genes		
	All Genes	PPE Genes	PE Genes	PPE Genes	PE Genes		Number	OR	Up-Regulated%
M1: Brown	256	17	37	1.54	1.45	69%	27	0.91	44%
M2: Pink	45	10	5	6.39	1.02	73%	3	0.55	33%
M3: Magenta	42	3	4	1.58	0.85	79%	3	0.59	67%
M4: Turquoise	726	26	79	0.65	0.99	33%	108	1.71	17%
M5: Blue	378	11	43	0.55	1.06	44%	37	0.82	46%
M6: Green	176	7	12	0.83	0.57	61%	21	1.06	62%
M7: Red	72	9	7	3.11	0.87	60%	8	0.97	29%
M8: Yellow	182	7	19	0.80	0.94	36%	12	0.53	42%
M9: Black	60	1	6	0.34	0.90	73%	1	0.13	100%
Σ	1937	91	212			47%	220		

For each gene module, the table shows the total number of genes, the number of predominantly placenta-expressed (PPE) genes, the number of genes differentially expressed in preterm preeclampsia [57] (PE), the odds ratios (OR) indicating enrichments in PPE and PE genes, the percentage of up-regulated genes, the number of transcription regulatory genes, their ORs, and the percentage of up-regulated genes among them.

## Supplementary Materials

Supplementary materials can be found at https://www.mdpi.com/1422-0067/21/2/628/s1.
