# Supplementary material for: Placenta-Specific Genes, Their Regulation During Villous Trophoblast Differentiation and Dysregulation in Preterm Preeclampsia
Source: Int J Mol Sci. 2020 Jan 17;21(2):628. doi: 10.3390/ijms21020628 (PMC7013556; doi:10.3390/ijms21020628)
Supplement: Supplementary file 1 [file ijms-21-00628-s001.zip › Supp_info_figures.pdf]

SUPPLEMENTARY INFORMATION

SUPPLEMENTARY MATERIALS AND METHODS

Experimental procedures

Human subjects and specimens, clinical definitions: Placentas (n=6) were collected at the Perinatology Research Branch of the *Eunice Kennedy Shriver* National Institute of Child Health and Human Development (NICHD), National Institutes of Health (NIH), Department of Health and Human Services (DHHS; Bethesda, MD, and Detroit, MI, USA) and Wayne State University (Detroit, MI, USA). All women provided written informed consent prior to the collection of samples, which was approved by the Institutional Review Boards of NICHD and Wayne State University. Placentas were taken from normal pregnant women delivering at term (≥37 weeks) who had no medical complications or clinical or histological signs of chorioamnionitis and delivered a neonate appropriate for gestational age [1].

Primary villous trophoblast cultures: Cytotrophoblast were isolated as published previously [2]. In brief, a 100g specimen of placental tissue was cut, rinsed in PBS, and digested sequentially with Trypsin (0.25%; Life Technologies, Grand Island, NY, USA) and DNase I (60U/ml; Sigma-Aldrich, St. Louis, MO, USA) for 90min at 37°C. Cells were then filtered through 100µm Falcon nylon mesh cell strainers (BD Biosciences, San Jose, CA, USA), and erythrocytes were lysed with NH_4Cl solution (Stemcell Technologies, Vancouver, BC, Canada). After washing, resuspended cells were layered over 20-50% Percoll gradients and then centrifuged for 20min at 1,200g. The bands that contained trophoblasts were collected, and non-trophoblastic cells were negatively selected with anti-CD9 (20µg/ml) and anti-CD14 (20µg/ml) mouse monoclonal antibodies (R&D Systems, Minneapolis, MN, USA), MACS anti-mouse IgG microbeads (Miltenyi Biotec, Auburn, CA, USA) and MS columns (Miltenyi Biotec). Primary trophoblasts were then plated on collagen-coated 12-well plates (BD Biosciences) at $3x10^6$ cells/well density in Iscove's modified Dulbecco's medium (IMDM; Life Technologies) supplemented with 10% fetal bovine serum and 1% penicillin/streptomycin (P/S). Subsequently, the effect of trophoblast differentiation on gene expression was tested by keeping primary trophoblasts in IMDM containing 5% non-pregnant human serum (SeraCare, Milford, MA, USA) and 1% P/S for 7 days. The medium was replenished every 24h, and cells were harvested for total RNA every 24h. All experiments were run in triplicate. Brightfield images of differentiating trophoblast cultures were taken on selected days with an CKX41 inverted microscope (Olympus Corp., Center Valley, PA, USA).

Total RNA isolation: Total RNA was isolated from primary trophoblasts harvested on Days 0-7 of differentiation with an RNeasy kit (QIAGEN, Valencia, CA, USA), according to the manufacturer's

recommendation. The 28S/18S ratios and the RNA integrity numbers were assessed using a Bioanalyzer 2100 (Agilent Technologies, Wilmington, DE, USA), and RNA concentrations were measured with NanoDrop1000 (Thermo Scientific, Wilmington, DE, USA).

Microarray: DNase-treated RNA samples (n=3; 500ng) were amplified and biotin-labeled with the Illumina TotalPrep RNA Amplification Kit (Ambion, LifeTechnologies, Foster City, CA, USA); the labeled cRNAs were then hybridized to HumanHT-12v4 Expression BeadChips (Illumina Inc., San Diego, CA, USA). Subsequently, BeadChips were imaged using an Illumina BeadArray Reader, and raw data were obtained with Illumina BeadStudio Software V.3.4.0.

qRT-PCR validation was performed for nine genes (three transcription factors involved in trophoblast differentiation, five differentially expressed target genes, and a housekeeping gene, see **Table S10**). Total RNA (n=5; 500ng) was reverse transcribed with a High Capacity cDNA Reverse Transcription Kit using random hexamers (Applied Biosystems, Foster City, CA, USA). TaqMan assays were used for gene expression profiling on the Biomark high-throughput qRT-PCR system (Fluidigm, San Francisco, CA, USA), according to the manufacturers' instructions.

Bioinformatic analyses

Microarray data analysis was performed in the R statistical language and environment. First, expression intensities were background-corrected using the default method in the *lumiR* function of the LUMI package [3–6]. Data were then variance-stabilized via $\log_2$ transformation and normalized with the *quantile* method. From the 47,307 probes on the array, we included those 25,025 probes (14,197 unique genes) into the subsequent steps of analysis that had at least two samples with a false discovery rate (FDR)-adjusted p-value of <0.1. Entrez IDs of the probes were determined using the *illuminaHumanv4.db* package. Non-annotated probes were removed from further analysis. Other information (e.g. chromosome location, gene symbol) was downloaded from the Entrez database.

Analysis of differentially expressed genes: The overall changes in gene expressions during the seven days of differentiation were analyzed using the *"limma"* package by comparing the mean expressions on a given day versus Day 0. The highest fold change for a given gene was defined as the maximum of the daily expression differences during the seven-day time period. Differentially expressed genes (n=1,937) were identified if they had a high fold change of ≥2 and FDR-adjusted p-values of <0.1 on at least one day of the time period. Differentially expressed gene enrichments among chromosomes, biological processes, cellular components, molecular functions, InterPro protein domains, KEGG and Reactome pathways, UniProt keywords, sequence features and tissues as well as transcription factor binding sites conserved in the human/mouse/rat alignment (UCSC_TFBS) were analyzed with the

DAVID Bioinformatics Resource. The significance was set at <0.2 according to FDR-adjustment based on the Benjamini-Hochberg method. The network of biological processes among differentially expressed genes was visualized with BiNGO [7].

The temporal changes in gene expression throughout the seven-day experiment were compared in two different ways. In the first analysis, we compared mean gene expressions on a given day versus Day 0 (**Figure 5A**). In the second analysis, we compared mean gene expressions on a given day versus the previous day (**Figure 5B**). In both analyses, the sets of differentially expressed genes were defined for each of the seven days.

Genes with a high change in expression were identified by analyzing the enrichment of predominantly placenta-expressed (PPE) genes within various gene sets according to log-fold change thresholds **(Figure S7, Table S5**). A hierarchical cluster analysis among high-expression-change genes was performed to identify gene expression change patterns during trophoblast differentiation. To avoid bias due to differences in baseline and maximum gene expression values, expression data were normalized by converting expression differences into a 0 to 1 scale. The clustering was based on Euclidean distance and complete linkage methods. The enrichments of the five gene clusters among chromosomes, biological processes, cellular components, molecular functions, InterPro protein domains, KEGG and Reactome pathways, UniProt keywords, sequence features and tissues as well as transcription factor binding sites conserved in the human/mouse/rat alignment (UCSC_TFBS) were analyzed with the DAVID Bioinformatics Resource. The significance was set at <0.2 according to FDR-adjustment based on the Benjamini-Hochberg method. The enrichments of the five clusters of genes among common regulators were tested using Pathway Studio 9.0 (Elsevier, Amsterdam, The Netherlands). Significance was set at p-values of <0.05.

Weighted gene co-expression network analysis (WGCNA) [8, 9] was performed on the 1,937 differentially expressed genes to identify distinct gene regulatory modules during trophoblast differentiation. First, a gene pair-wise similarity matrix was computed, which was then soft-thresholded by raising it to the power of 10 to calculate the adjacency matrix. From the adjacency matrix, we calculated the unsigned topology overlap matrix (TOM). Distance matrix was defined as 1-TOM and used for average linkage hierarchical clustering. Gene modules identified with this approach were further tested for their enrichments for PPE genes and up-regulated genes using the Fisher's exact test. Moreover, the enrichments of the gene modules among chromosomes, biological processes, cellular components, molecular functions, InterPro protein domains, KEGG and Reactome pathways, UniProt keywords, sequence features and tissues as well as transcription factor binding sites conserved in the human/mouse/rat alignment (UCSC_TFBS) were analyzed with the DAVID Bioinformatics Resource.

Identification of predominantly placenta-expressed genes: The human BioGPS microarray data on 79 human tissues, cells, and cell lines [10] were downloaded to search for PPE genes. The probesets (Affymetrix, Santa Clara, CA, USA) were defined as having predominant placental expression if their placental expression was 1) ≥1,000 fluorescence units; 2) ≥6 fold higher than the median values for the 78 other tissue and cell sources; and 3) ≥2 fold higher than their expression in the tissue with the second highest expression. The resulting 215 probesets corresponded to 153 unique genes, while 11 additional genes not present on the Affymetrix microarray platform were added to this list based on previously published evidence [11, 12].

Functional analysis of predominantly placenta-expressed genes: Transcription regulatory gene products (transcription factors and co-factors) were identified in the Uniprot (www.uniprot.org) and Gene Ontology (www.geneontology.org) databases using the Uniprot Keyword "transcription regulation" (KW-0805) as well as the GO terms "transcription factor activity, sequence-specific DNA binding" (GO:0003700) and "transcription corepressor activity" (GO:0003714). The enrichment of PPE genes among chromosomes, biological processes, cellular components, molecular functions, InterPro protein domains, KEGG and Reactome pathways, Protein Information Resource superfamilies and keywords, UniProt sequence features and tissues were analyzed with the PANTHER Classification System [13] via Gene Ontology and with the DAVID Bioinformatics Resource [14, 15]. Network connections of PPE genes were retrieved from previously published evidence [2, 16, 17] and by using the Pathway Studio 9.0 software, which utilizes the ResNet Mammalian Database 5.0, an inclusive set of pathways, cell processes, and molecular interactions of proteins and small molecules uniquely representing relevant information from the entirety of PubMed (www.ariadnegenomics.com).

Chromosomal localization of predominantly placenta-expressed genes: The chromosomal localization of PPE genes was visualized by Circos [18]. The chromosomal enrichment of PPE genes was calculated using the Fisher's exact test.

Placental localization and expression pattern of predominantly placenta-expressed genes: The Human Protein Atlas database (www.proteinatlas.org) and the GeneCards (www.genecards.org) records, the latter being based on the Uniprot and Gene Ontology cellular component databases, were used to analyze the tissue localization and expression pattern of PPE genes in the human placenta. The expression of PPE genes in the extravillous trophoblast and villous trophoblast lineages were analyzed by retrieving three earlier published microarray datasets [19–21] and reanalyzing expression data for PPE genes. Raw Affymetrix GeneChip Human Genome U133A 2.0 Array data from Bilban *et al.* [20] and Tilburgs *et al.* [21] were downloaded from GEO (GSE9773) and

ArrayExpress (E-MATB-3217) and processed with the AFFY [22] and LIMMA [23] packages. We used RMA normalization and then calculated the log-fold change only for PPE genes. In the case of the dataset of Apps *et al.* [19], processed data from Illumina Human HT-12 V3 BeadArrays were downloaded from ArrayExpress (E-MATB-429) and the log-fold change for PPE genes was calculated. Data were visualized by heat maps and bar charts.

Determining transcriptional networks from DNase footprinting data: To determine transcriptional regulatory networks, we used genome-wide maps of *in vivo* DNaseI footprints to identify occupied binding sites of sequence-specific transcription factors, following the method of Neph *et al.* [24] Two DNaseI footprinting data sets were used: a data set obtained from H1 human embryonic stem cells differentiated into trophoblast cells by BMP4 treatment (ENCODE accession ENCSR179CDH), and a data set obtained from human amniotic epithelial cells (HAEpiC; GEO accession GSM646560). The latter data set was included because of its higher sequencing depth and resulting better coverage.

For the trophoblast footprinting, hotspots (regions with high sensitivity to cleavage by DNaseI) were identified from read alignments by the hotspot2 program by Eric Rynes (github.com/Altius/hotspot2) using an FDR of <0.01. For the HAEpiC data set, the hotspots data file was downloaded from the GEO database (accession GSM646560). For both data sets, footprints were identified with the footprinting2012 program by Neph [25] (github.com/sjneph/footprinting2012). For the footprints, a flanking region between 3 and 10 bases and a center region between 6 and 40 bases were required, and footprints with an occupancy score of <0.95 within a hotspot were accepted. Human transcription factor lists and binding motif position weight matrices were downloaded from The Human Transcription Factors website (humantfs.ccbr.utoronto.ca) [26]. After filtering for high-quality evidence for transcription factor function, 1,317 transcription factors were retained along with the binding site motif marked as "best" in the database. The FIMO program [27] was used to find transcription factor binding sites within hotspots; a p-value threshold of 10^{-5} was set. Transcription factor binding sites overlapping with a footprint were identified in the putative regulatory region of all genes, defined as being within 5 kb upstream and 1 kb downstream of the transcription start site (TSS). Gene annotations were taken from the refFlat tables of the UCSC Genome Browser [28] (hg19 assembly for the HAEpiC data and hg38 for the trophoblast data, respectively). As >90% of the identified transcription binding sites were found to be in known promoter or enhancer regions based on the GeneHancer database [29], no further filtering of the binding regions was performed.

SUPPLEMENTARY FIGURE LEGENDS

Figure S1. Flow-chart of experimental procedures and analytical methods studying normal villous trophoblast development. The flow-chart depicts the data, databases, experimental and bioinformatics methods used for the study as well as their outputs and sequential order (arrows). Abbreviations: DE, differentially expressed; EVT, extravillous trophoblast; HEC, high expression change; PPE, predominantly placental expressed; VT, villous trophoblast; WGCNA, weighted gene co-expression network analysis.

Figure S2. Expression of predominantly placental expressed genes in villous trophoblasts compared to extravillous trophoblasts. Microarray data from three independent datasets [19–21] **(a, b, c)** was reanalyzed. Color codes depict $\log_2$ gene expression ratios. Red indicates higher gene expression in extravillous trophoblasts (EVTs), while blue depicts higher gene expression in villous trophoblasts (VTs). Grey is used to indicate missing data.

Figure S3. Microarray validation with qRT-PCR. Scatter plots showing correlations between microarray intensity and qRT-PCR expression data. Points depict respective microarray (n=3) and qRT-PCR (n=3) data from Day 0 to Day 7 during trophoblast differentiation. **a)** Data for PPE genes encoding transcription regulatory proteins. The average Pearson correlation for these three genes was 0.834. **b)** Data for PPE genes encoding secreted trophoblastic proteins. The average Pearson correlation for these five genes was 0.972.

Figure S4. Volcano plot analysis of microarray data. Microarray analysis of gene expression profiles throughout the 7-day differentiation period was performed in two different ways. **A)** On the left panel, volcano plots show differential gene expression between a given day versus Day 0. **B)** On the right panel, volcano plots show differential gene expression between a given day versus the previous day. Dots in the upper right (red, up-regulated) and left (blue, down-regulated) quadrants represent genes with a fold change of ≥2 and a false discovery rate corrected p-value of < 0.1.

Figure S5. Biological characteristics of gene modules (M) among differentially expressed genes during villous trophoblast differentiation. A) Gene module enrichments for UniProt tissues are shown with bar-charts. **B)** Gene module enrichments for cellular components are shown with bar-charts.

Figure S6. Time course of gene expression during villous trophoblast differentiation. The time course of expression changes, measured as $\log_2$ fold change, of genes in the 9 gene modules defined by WGCNA (Fig. 4). Plots for placental modules are shown in blue, and those for non-placental modules in red.

Figure S7. Identification of genes with high expression change during villous trophoblast differentiation. A) The enrichment of predominantly placental expressed (PPE) genes among sets of differentially expressed (DE) genes was analyzed according to log_2-fold change thresholds. Bar charts depict PPE gene enrichment among genes with equal or higher differential expression than the given log_2-fold change threshold; the darker red color indicates the difference from the previous threshold. The continuous increase in enrichment with higher log_2−fold change thresholds rises at 2.5; therefore, genes with ≥2.5 log_2-fold change were defined as genes with high expression change (HEC). **B)** The bar chart shows the percentage of all genes as the function of log_2-fold change thresholds. HEC genes include ~1.4% of all genes with valid datapoint in the microarray. The red bar depicts 1.5%.

Figure S8. Chromosomal distribution of differentially expressed genes during villous trophoblast differentiation. A) Bar charts show the number of differentially expressed genes (DE, green) and genes with high expression change (HEC, yellow) among DE genes on various chromosomes. There was no enrichment of DE genes for any chromosomes, while HEC genes were enriched on Chr19 (OR=1.91, p=0.0028). **B)** Bar charts show the number of HEC genes (yellow) and the intersection of these genes with predominantly placental expressed genes (PPE, red) on various chromosomes. HEC PPE genes were over-represented on Chr19 (OR=12.17, p<0.0001) and on Chr17 (OR=2.76, p=0.0368).

Figure S9. Venn diagram of the various gene sets defined in this paper. The diagram helps clarify the relationships among the gene sets.

SUPPLEMENTARY TABLE LEGENDS

Table S1. Predominantly placental expressed genes. The table lists 164 predominantly placental expressed genes and their chromosomal location.

Table S2. Enrichments among predominantly placental expressed genes. The table lists 164 predominantly placental expressed genes and their enrichments among chromosomes, biological processes, cellular components, molecular functions, InterPro protein domains, KEGG and Reactome pathways, Protein Information Resource superfamilies and keywords, UniProt sequence features and tissues. Enrichments were analyzed with the DAVID Bioinformatics Resource [14, 15]. The significance threshold was set at FDR<0.2 according to adjustment with the Benjamini-Hochberg method. Significant enrichments are highlighted with yellow.

Table S3. Predominantly placental expressed gene expression in trophoblastic lineages. The table lists 164 predominantly placental expressed genes and their expression in extravillous, villous or both trophoblastic lineages. The data is based on the reanalysis of three microarray datasets published in Refs [19–21].

Table S4. Differentially expressed genes. The table lists 1,937 differentially expressed (DE) genes and defines their position in modules and on chromosomes (CHR) as well as among predominantly placental expressed (PPE) genes, preeclampsia-associated (PE) genes, transcription regulatory (TR) genes, and high expression change (HEC) genes. Differential expression is depicted with log_2-fold change values, p values, and FDR-adjusted p values. The enrichments of DE genes among chromosomes, biological processes, cellular components, molecular functions, InterPro protein domains, KEGG and Reactome pathways, UniProt keywords, sequence features, and tissues as well as transcription factor binding sites conserved in the human/mouse/rat alignment (UCSC_TFBS) are also shown. Enrichments were analyzed with the DAVID Bioinformatics Resource [14, 15]. The significance threshold was set at FDR<0.2 according to adjustment with the Benjamini-Hochberg method. Significant enrichments are highlighted with yellow.

Table S5. Gene module enrichments. The table separately lists genes in the M1-M2-M3-M7 as well as the M4-M5-M6-M8-M9 modules. The enrichments of the nine modules as well as the combination of M1-M2-M3-M7 or M4-M5-M6-M8-M9 modules among chromosomes, biological processes, cellular components, molecular functions, InterPro protein domains, KEGG and Reactome pathways, UniProt keywords, sequence features and tissues as well as transcription factor binding sites conserved in the human/mouse/rat alignment (UCSC_TFBS) were also depicted. Enrichments were analyzed with the DAVID Bioinformatics Resource [14, 15]. The significance threshold was

set at pFDR<0.2 according to adjustment with the Benjamini-Hochberg method. Significant enrichments are highlighted with yellow.

Table S6. iPathwayGuide Analysis. Pathways identified as impacted by the Impact Analysis method [30–32]. Significant pathways with an FDR-corrected p-value < 0.05 are highlighted in bold.

Table 7. Enrichments among high expression gene clusters. The table lists 204 genes with high expression change (HEC) among differentially expressed genes and their membership in five clusters (C1-C5). The enrichments of the five clusters among chromosomes, biological processes, cellular components, molecular functions, InterPro protein domains, KEGG and Reactome pathways, UniProt keywords, sequence features, and tissues as well as transcription factor binding sites conserved in the human/mouse/rat alignment (UCSC_TFBS) were also depicted. Enrichments were analyzed with the DAVID Bioinformatics Resource [14, 15]. The significance threshold was set at FDR<0.2 according to adjustment with the Benjamini-Hochberg method. Significant enrichments are highlighted with yellow.

Table 8. Regulators of high expression change gene clusters. The table lists 204 genes with high expression change (HEC) among differentially expressed genes and their membership in five clusters (C1-C5). The enrichments of the five clusters among common regulators were tested using Pathway Studio 9.0. The significance was set at p-values of <0.05 and overlapping values of 2. As default, the software generated only the top 1000 significant common regulators for clusters C2-4.

Table S9. Transcription factors regulating villous trophoblast differentiation. Transcription factors identified from DNaseI footprinting data as binding to promoter regions of DE genes are listed. DE transcription factors are shown in uppercase letters. For each factor, the fraction of its targets among placental-module and non-placental-module genes, as well as the ratio of the two fractions, i.e., the enrichment among the placental-module-genes are shown.

Table S10. TaqMan assays. The table lists the TaqMan assays used for gene expression profiling.

References

1. Alexander GR, Himes JH, Kaufman RB, et al (1996) A United States national reference for fetal growth. Obstet Gynecol 87:163–168. https://doi.org/10.1016/0029-7844(95)00386-X

2. Than NG, Romero R, Xu Y, et al (2014) Evolutionary origins of the placental expression of chromosome 19 cluster galectins and their complex dysregulation in preeclampsia. Placenta 35:855–865. https://doi.org/10.1016/j.placenta.2014.07.015

3. Du P, Kibbe WA, Lin SM (2008) lumi: a pipeline for processing Illumina microarray. Bioinforma Oxf Engl 24:1547–1548. https://doi.org/10.1093/bioinformatics/btn224

4. Du P, Zhang X, Huang C-C, et al (2010) Comparison of Beta-value and M-value methods for quantifying methylation levels by microarray analysis. BMC Bioinformatics 11:587. https://doi.org/10.1186/1471-2105-11-587

5. Lin SM, Du P, Huber W, Kibbe WA (2008) Model-based variance-stabilizing transformation for Illumina microarray data. Nucleic Acids Res 36:e11. https://doi.org/10.1093/nar/gkm1075

6. Du P, Kibbe WA, Lin SM (2007) nuID: a universal naming scheme of oligonucleotides for illumina, affymetrix, and other microarrays. Biol Direct 2:16. https://doi.org/10.1186/1745-6150-2-16

7. Maere S, Heymans K, Kuiper M (2005) BiNGO: a Cytoscape plugin to assess overrepresentation of gene ontology categories in biological networks. Bioinforma Oxf Engl 21:3448–3449. https://doi.org/10.1093/bioinformatics/bti551

8. Zhang B, Horvath S (2005) A general framework for weighted gene co-expression network analysis. Stat Appl Genet Mol Biol 4:Article17. https://doi.org/10.2202/1544-6115.1128

9. Langfelder P, Horvath S (2008) WGCNA: an R package for weighted correlation network analysis. BMC Bioinformatics 9:559. https://doi.org/10.1186/1471-2105-9-559

10. Su AI, Wiltshire T, Batalov S, et al (2004) A gene atlas of the mouse and human protein-encoding transcriptomes. Proc Natl Acad Sci U S A 101:6062–6067. https://doi.org/10.1073/pnas.0400782101

11. Rawn SM, Cross JC (2008) The evolution, regulation, and function of placenta-specific genes. Annu Rev Cell Dev Biol 24:159–181. https://doi.org/10.1146/annurev.cellbio.24.110707.175418

12. Than NG, Romero R, Goodman M, et al (2009) A primate subfamily of galectins expressed at the maternal-fetal interface that promote immune cell death. Proc Natl Acad Sci U S A 106:9731–9736. https://doi.org/10.1073/pnas.0903568106

13. Mi H, Muruganujan A, Casagrande JT, Thomas PD (2013) Large-scale gene function analysis with the PANTHER classification system. Nat Protoc 8:1551–1566. https://doi.org/10.1038/nprot.2013.092

14. Huang DW, Sherman BT, Lempicki RA (2009) Bioinformatics enrichment tools: paths toward the comprehensive functional analysis of large gene lists. Nucleic Acids Res 37:1–13. https://doi.org/10.1093/nar/gkn923

15. Huang DW, Sherman BT, Lempicki RA (2009) Systematic and integrative analysis of large gene lists using DAVID bioinformatics resources. Nat Protoc 4:44–57. https://doi.org/10.1038/nprot.2008.211

16. Lichtner B, Knaus P, Lehrach H, Adjaye J (2013) BMP10 as a potent inducer of trophoblast differentiation in human embryonic and induced pluripotent stem cells. Biomaterials 34:9789–9802. https://doi.org/10.1016/j.biomaterials.2013.08.084

17. Cheng Y-H, Aronow BJ, Hossain S, et al (2004) Critical role for transcription factor AP-2alpha in human trophoblast differentiation. Physiol Genomics 18:99–107. https://doi.org/10.1152/physiolgenomics.00181.2003

18. Krzywinski M, Schein J, Birol I, et al (2009) Circos: an information aesthetic for comparative genomics. Genome Res 19:1639–1645. https://doi.org/10.1101/gr.092759.109

19. Apps R, Sharkey A, Gardner L, et al (2011) Genome-wide expression profile of first trimester villous and extravillous human trophoblast cells. Placenta 32:33–43. https://doi.org/10.1016/j.placenta.2010.10.010

20. Bilban M, Haslinger P, Prast J, et al (2009) Identification of novel trophoblast invasion-related genes: heme oxygenase-1 controls motility via peroxisome proliferator-activated receptor gamma. Endocrinology 150:1000–1013. https://doi.org/10.1210/en.2008-0456

21. Tilburgs T, Crespo ÂC, van der Zwan A, et al (2015) Human HLA-G+ extravillous trophoblasts: Immune-activating cells that interact with decidual leukocytes. Proc Natl Acad Sci U S A 112:7219–7224. https://doi.org/10.1073/pnas.1507977112

22. Gautier L, Cope L, Bolstad BM, Irizarry RA (2004) affy--analysis of Affymetrix GeneChip data at the probe level. Bioinforma Oxf Engl 20:307–315. https://doi.org/10.1093/bioinformatics/btg405

23. Ritchie ME, Phipson B, Wu D, et al (2015) limma powers differential expression analyses for RNA-sequencing and microarray studies. Nucleic Acids Res 43:e47. https://doi.org/10.1093/nar/gkv007

24. Neph S, Stergachis AB, Reynolds A, et al (2012) Circuitry and dynamics of human transcription factor regulatory networks. Cell 150:1274–1286. https://doi.org/10.1016/j.cell.2012.04.040

25. Neph S, Vierstra J, Stergachis AB, et al (2012) An expansive human regulatory lexicon encoded in transcription factor footprints. Nature 489:83–90. https://doi.org/10.1038/nature11212

26. Lambert SA, Jolma A, Campitelli LF, et al (2018) The Human Transcription Factors. Cell 172:650–665. https://doi.org/10.1016/j.cell.2018.01.029

27. Grant CE, Bailey TL, Noble WS (2011) FIMO: scanning for occurrences of a given motif. Bioinforma Oxf Engl 27:1017–1018. https://doi.org/10.1093/bioinformatics/btr064

28. Haeussler M, Zweig AS, Tyner C, et al (2019) The UCSC Genome Browser database: 2019 update. Nucleic Acids Res 47:D853–D858. https://doi.org/10.1093/nar/gky1095

29. Fishilevich S, Nudel R, Rappaport N, et al (2017) GeneHancer: genome-wide integration of enhancers and target genes in GeneCards. Database J Biol Databases Curation 2017:. https://doi.org/10.1093/database/bax028

30. Draghici S, Khatri P, Tarca AL, et al (2007) A systems biology approach for pathway level analysis. Genome Res 17:1537–1545. https://doi.org/10.1101/gr.6202607

31. Tarca AL, Draghici S, Khatri P, et al (2009) A novel signaling pathway impact analysis. Bioinforma Oxf Engl 25:75–82. https://doi.org/10.1093/bioinformatics/btn577

32. Khatri P, Draghici S, Tarca AL, et al (2007) A System Biology Approach for the Steady-State Analysis of Gene Signaling Networks. In: Rueda L, Mery D, Kittler J (eds) Progress in Pattern Recognition, Image Analysis and Applications. Springer Berlin Heidelberg, Berlin, Heidelberg, pp 32–41

Figure S1

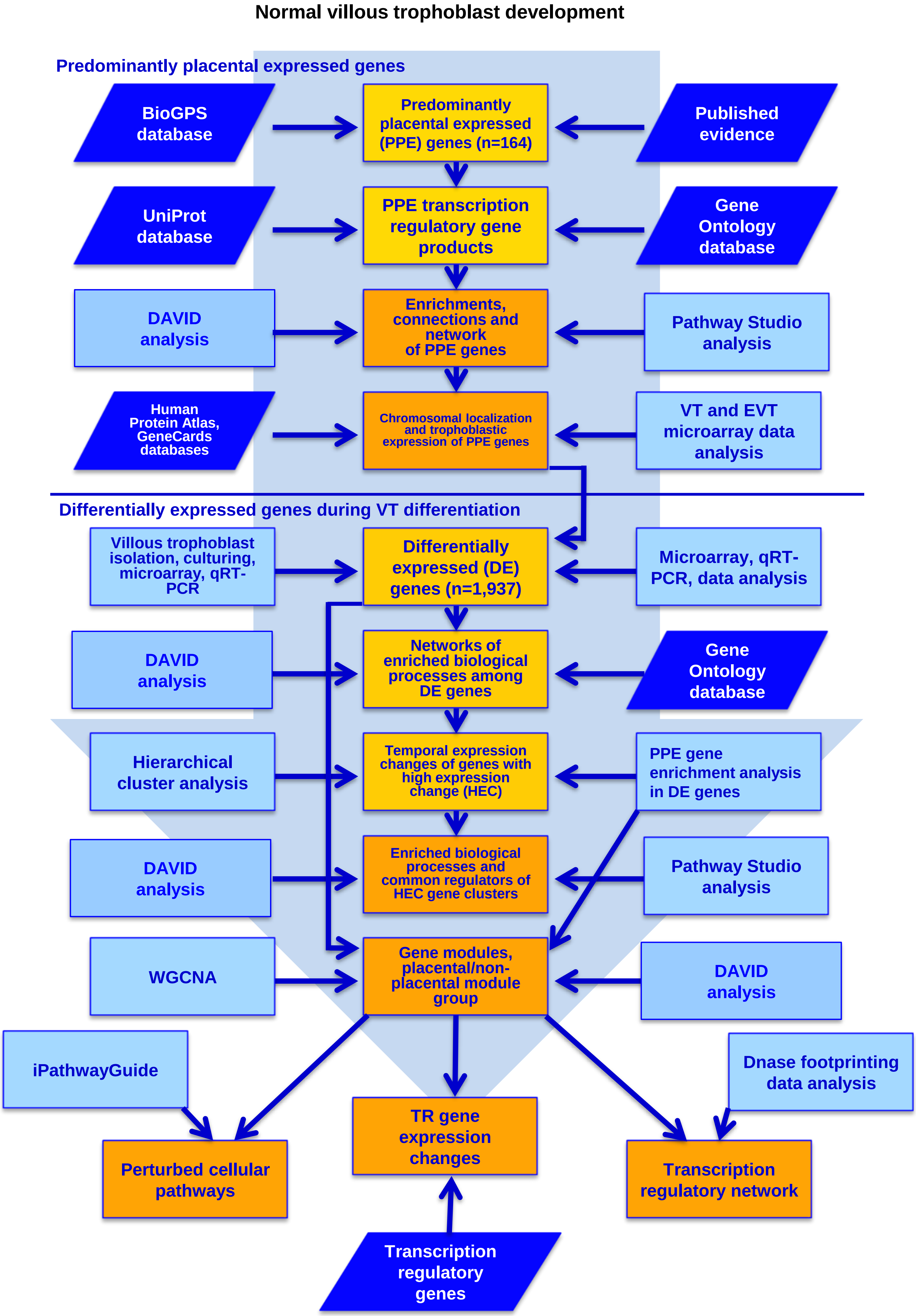
Normal villous trophoblast development
Predominantly placental expressed genes
BioGPS database
Predominantly placental expressed (PPE) genes (n=164)
Published evidence
UniProt database
PPE transcription regulatory gene products
Gene Ontology database
DAVID analysis
Enrichments, connections and network of PPE genes
Pathway Studio analysis
Human Protein Atlas, GeneCards databases
Chromosomal localization and trophoblastic expression of PPE genes
VT and EVT microarray data analysis
Differentially expressed genes during VT differentiation
Villous trophoblast isolation, culturing, microarray, qRT-PCR
Differentially expressed (DE) genes (n=1,937)
Microarray, qRT-PCR, data analysis
DAVID analysis
Networks of enriched biological processes among DE genes
Gene Ontology database
Hierarchical cluster analysis
Temporal expression changes of genes with high expression change (HEC)
PPE gene enrichment analysis in DE genes
DAVID analysis
Enriched biological processes and common regulators of HEC gene clusters
Pathway Studio analysis
WGCNA
Gene modules, placental/non-placental module group
DAVID analysis
iPathwayGuide
Dnase footprinting data analysis
Perturbed cellular pathways
TR gene expression changes
Transcription regulatory network
Transcription regulatory genes

Figure S2

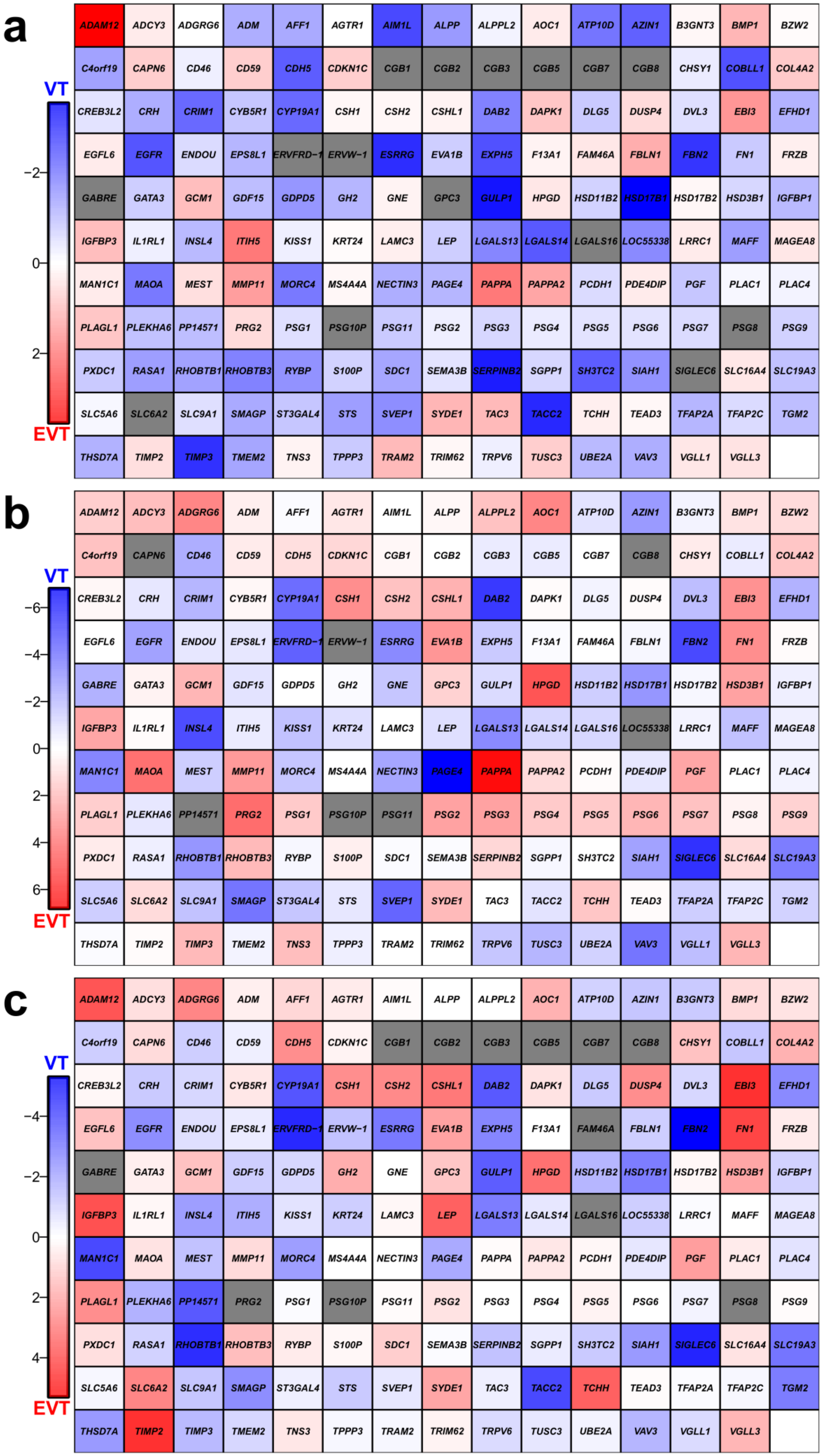
a
VT
−2
0
2
EVT
ADAM12 ADCY3 ADGRG6 ADM AFF1 AGTR1 AIM1L ALPP ALPPL2 AOC1 ATP10D AZIN1 B3GNT3 BMP1 BZW2
C4orf19 CAPN6 CD46 CD59 CDH5 CDKN1C CGB1 CGB2 CGB3 CGB5 CGB7 CGB8 CHSY1 COBLL1 COL4A2
CREB3L2 CRH CRIM1 CYB5R1 CYP19A1 CSH1 CSH2 CSHL1 DAB2 DAPK1 DLG5 DUSP4 DVL3 EBI3 EFHD1
EGFL6 EGFR ENDOU EPS8L1 ERVFRD−1 ERVW−1 ESRRG EVA1B EXPH5 F13A1 FAM46A FBLN1 FBN2 FN1 FRZB
GABRE GATA3 GCM1 GDF15 GDPD5 GH2 GNE GPC3 GULP1 HPGD HSD11B2 HSD17B1 HSD17B2 HSD3B1 IGFBP1
IGFBP3 IL1RL1 INSL4 ITIH5 KISS1 KRT24 LAMC3 LEP LGALS13 LGALS14 LGALS16 LOC55338 LRRC1 MAFF MAGEA8
MAN1C1 MAOA MEST MMP11 MORC4 MS4A4A NECTIN3 PAGE4 PAPPA PAPPA2 PCDH1 PDE4DIP PGF PLAC1 PLAC4
PLAGL1 PLEKHA6 PP14571 PRG2 PSG1 PSG10P PSG11 PSG2 PSG3 PSG4 PSG5 PSG6 PSG7 PSG8 PSG9
PXDC1 RASA1 RHOBTB1 RHOBTB3 RYBP S100P SDC1 SEMA3B SERPINB2 SGPP1 SH3TC2 SIAH1 SIGLEC6 SLC16A4 SLC19A3
SLC5A6 SLC6A2 SLC9A1 SMAGP ST3GAL4 STS SVEP1 SYDE1 TAC3 TACC2 TCHH TEAD3 TFAP2A TFAP2C TGM2
THSD7A TIMP2 TIMP3 TMEM2 TNS3 TPPP3 TRAM2 TRIM62 TRPV6 TUSC3 UBE2A VAV3 VGLL1 VGLL3
b
VT
−6
−4
−2
0
2
4
6
EVT
ADAM12 ADCY3 ADGRG6 ADM AFF1 AGTR1 AIM1L ALPP ALPPL2 AOC1 ATP10D AZIN1 B3GNT3 BMP1 BZW2
C4orf19 CAPN6 CD46 CD59 CDH5 CDKN1C CGB1 CGB2 CGB3 CGB5 CGB7 CGB8 CHSY1 COBLL1 COL4A2
CREB3L2 CRH CRIM1 CYB5R1 CYP19A1 CSH1 CSH2 CSHL1 DAB2 DAPK1 DLG5 DUSP4 DVL3 EBI3 EFHD1
EGFL6 EGFR ENDOU EPS8L1 ERVFRD−1 ERVW−1 ESRRG EVA1B EXPH5 F13A1 FAM46A FBLN1 FBN2 FN1 FRZB
GABRE GATA3 GCM1 GDF15 GDPD5 GH2 GNE GPC3 GULP1 HPGD HSD11B2 HSD17B1 HSD17B2 HSD3B1 IGFBP1
IGFBP3 IL1RL1 INSL4 ITIH5 KISS1 KRT24 LAMC3 LEP LGALS13 LGALS14 LGALS16 LOC55338 LRRC1 MAFF MAGEA8
MAN1C1 MAOA MEST MMP11 MORC4 MS4A4A NECTIN3 PAGE4 PAPPA PAPPA2 PCDH1 PDE4DIP PGF PLAC1 PLAC4
PLAGL1 PLEKHA6 PP14571 PRG2 PSG1 PSG10P PSG11 PSG2 PSG3 PSG4 PSG5 PSG6 PSG7 PSG8 PSG9
PXDC1 RASA1 RHOBTB1 RHOBTB3 RYBP S100P SDC1 SEMA3B SERPINB2 SGPP1 SH3TC2 SIAH1 SIGLEC6 SLC16A4 SLC19A3
SLC5A6 SLC6A2 SLC9A1 SMAGP ST3GAL4 STS SVEP1 SYDE1 TAC3 TACC2 TCHH TEAD3 TFAP2A TFAP2C TGM2
THSD7A TIMP2 TIMP3 TMEM2 TNS3 TPPP3 TRAM2 TRIM62 TRPV6 TUSC3 UBE2A VAV3 VGLL1 VGLL3
c
VT
−4
−2
0
2
4
EVT
ADAM12 ADCY3 ADGRG6 ADM AFF1 AGTR1 AIM1L ALPP ALPPL2 AOC1 ATP10D AZIN1 B3GNT3 BMP1 BZW2
C4orf19 CAPN6 CD46 CD59 CDH5 CDKN1C CGB1 CGB2 CGB3 CGB5 CGB7 CGB8 CHSY1 COBLL1 COL4A2
CREB3L2 CRH CRIM1 CYB5R1 CYP19A1 CSH1 CSH2 CSHL1 DAB2 DAPK1 DLG5 DUSP4 DVL3 EBI3 EFHD1
EGFL6 EGFR ENDOU EPS8L1 ERVFRD−1 ERVW−1 ESRRG EVA1B EXPH5 F13A1 FAM46A FBLN1 FBN2 FN1 FRZB
GABRE GATA3 GCM1 GDF15 GDPD5 GH2 GNE GPC3 GULP1 HPGD HSD11B2 HSD17B1 HSD17B2 HSD3B1 IGFBP1
IGFBP3 IL1RL1 INSL4 ITIH5 KISS1 KRT24 LAMC3 LEP LGALS13 LGALS14 LGALS16 LOC55338 LRRC1 MAFF MAGEA8
MAN1C1 MAOA MEST MMP11 MORC4 MS4A4A NECTIN3 PAGE4 PAPPA PAPPA2 PCDH1 PDE4DIP PGF PLAC1 PLAC4
PLAGL1 PLEKHA6 PP14571 PRG2 PSG1 PSG10P PSG11 PSG2 PSG3 PSG4 PSG5 PSG6 PSG7 PSG8 PSG9
PXDC1 RASA1 RHOBTB1 RHOBTB3 RYBP S100P SDC1 SEMA3B SERPINB2 SGPP1 SH3TC2 SIAH1 SIGLEC6 SLC16A4 SLC19A3
SLC5A6 SLC6A2 SLC9A1 SMAGP ST3GAL4 STS SVEP1 SYDE1 TAC3 TACC2 TCHH TEAD3 TFAP2A TFAP2C TGM2
THSD7A TIMP2 TIMP3 TMEM2 TNS3 TPPP3 TRAM2 TRIM62 TRPV6 TUSC3 UBE2A VAV3 VGLL1 VGLL3

Figure S3

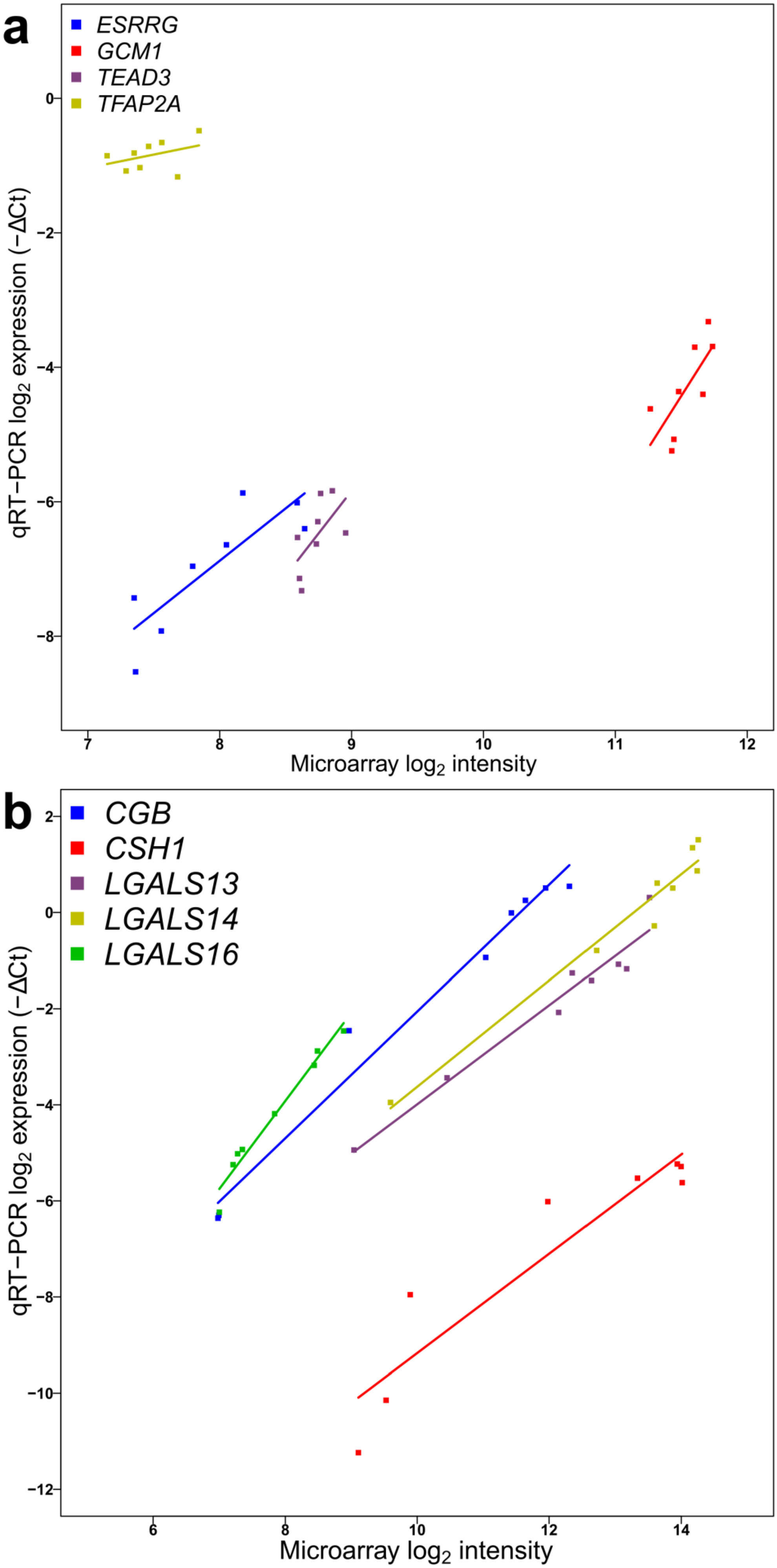
a
ESRRG
GCM1
TEAD3
TFAP2A
qRT−PCR log2 expression (−ΔCt)
Microarray log2 intensity
0
−2
−4
−6
−8
7
8
9
10
11
12
b
CGB
CSH1
LGALS13
LGALS14
LGALS16
2
0
−2
−4
−6
−8
−10
−12
6
8
10
12
14

Figure S4

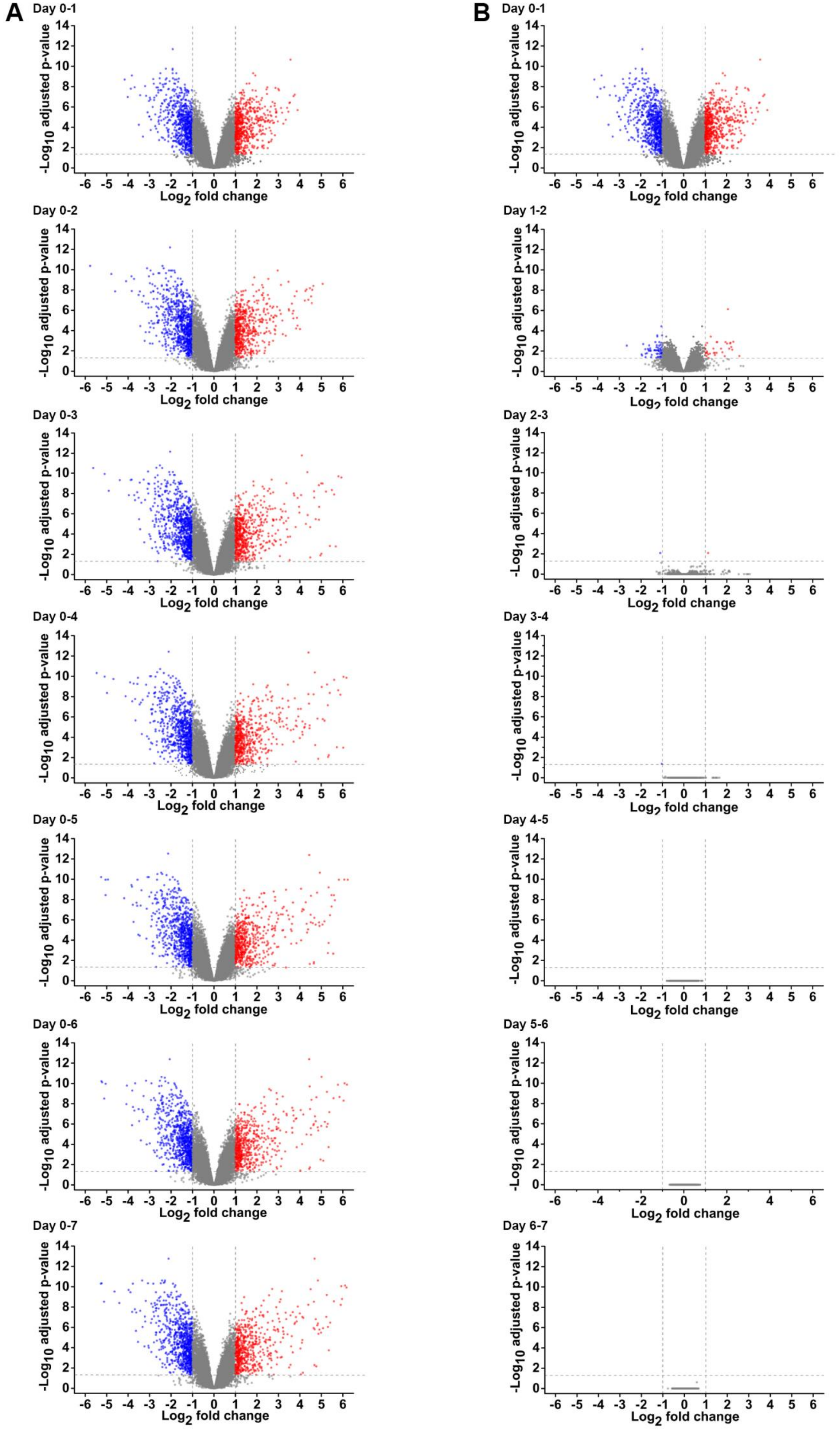
A
B
Day 0-1
Day 0-2
Day 0-3
Day 0-4
Day 0-5
Day 0-6
Day 0-7
Day 1-2
Day 2-3
Day 3-4
Day 4-5
Day 5-6
Day 6-7
-Log10 adjusted p-value
Log2 fold change

Figure S5

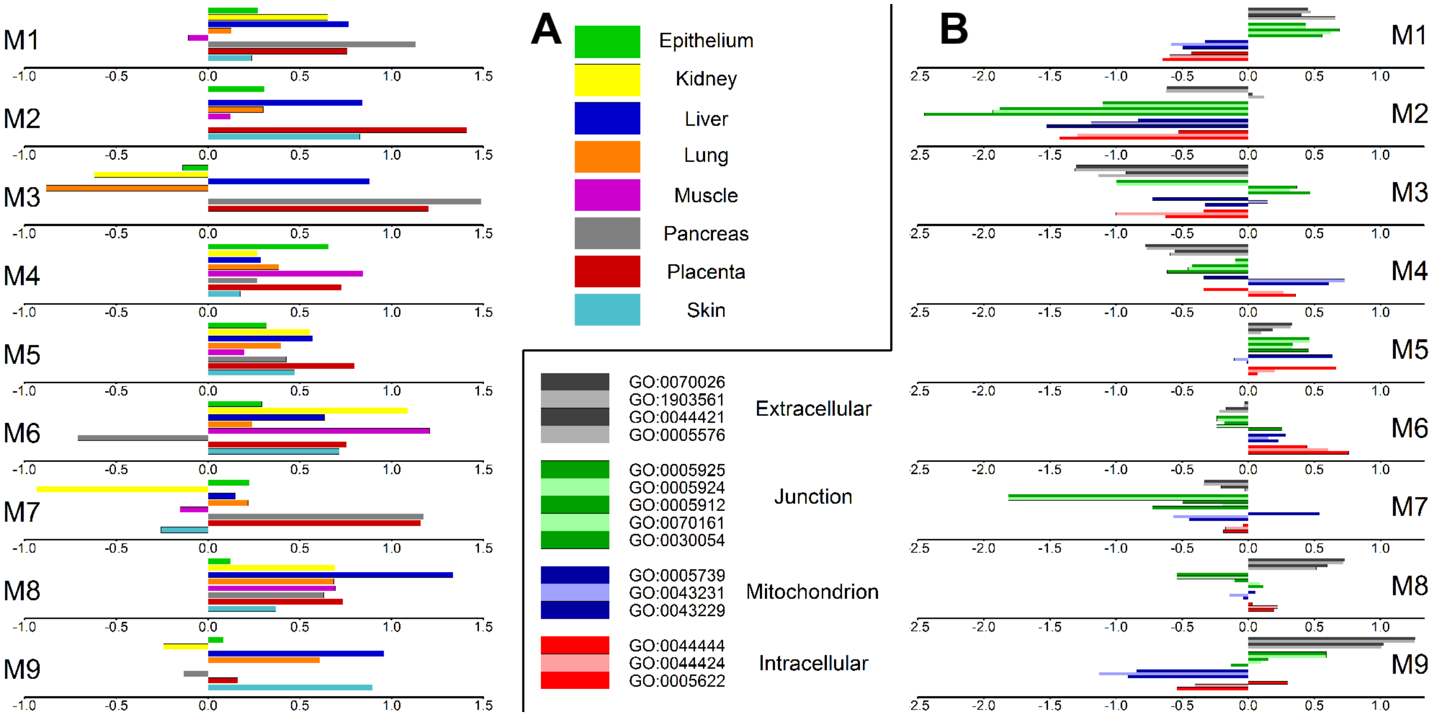

A
B
M1
M2
M3
M4
M5
M6
M7
M8
M9
Epithelium
Kidney
Liver
Lung
Muscle
Pancreas
Placenta
Skin
GO:0070026
GO:1903561
GO:0044421
GO:0005576
Extracellular
GO:0005925
GO:0005924
GO:0005912
GO:0070161
GO:0030054
Junction
GO:0005739
GO:0043231
GO:0043229
Mitochondrion
GO:0044444
GO:0044424
GO:0005622
Intracellular
-1.0
-0.5
0.0
0.5
1.0
1.5
2.5
-2.0
-1.5

Figure S6

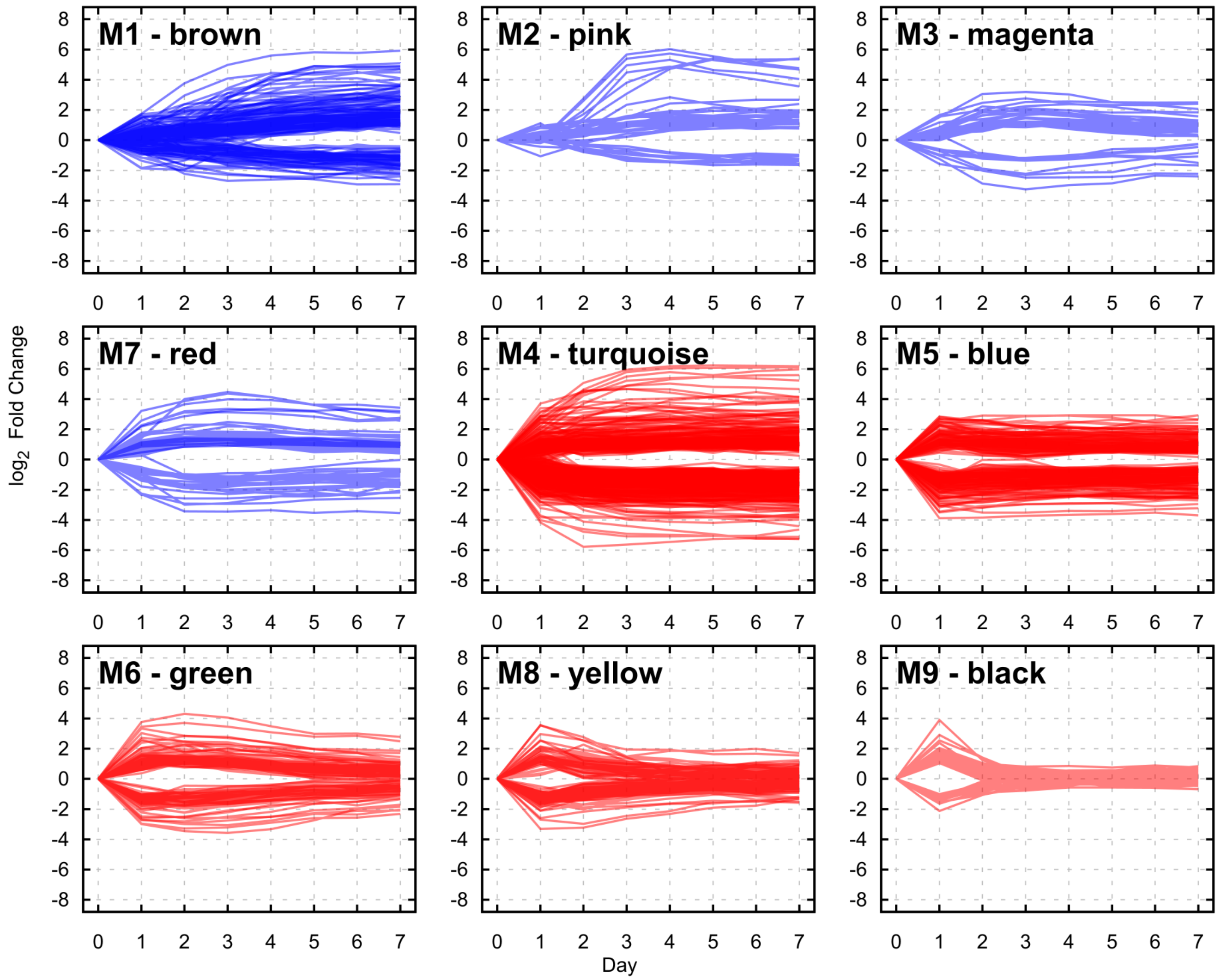
M1 - brown
M2 - pink
M3 - magenta
M7 - red
M4 - turquoise
M5 - blue
M6 - green
M8 - yellow
M9 - black
log2 Fold Change
Day
8
6
4
2
0
-2
-4
-6
-8
0
1
2
3
4
5
6
7

Figure S7

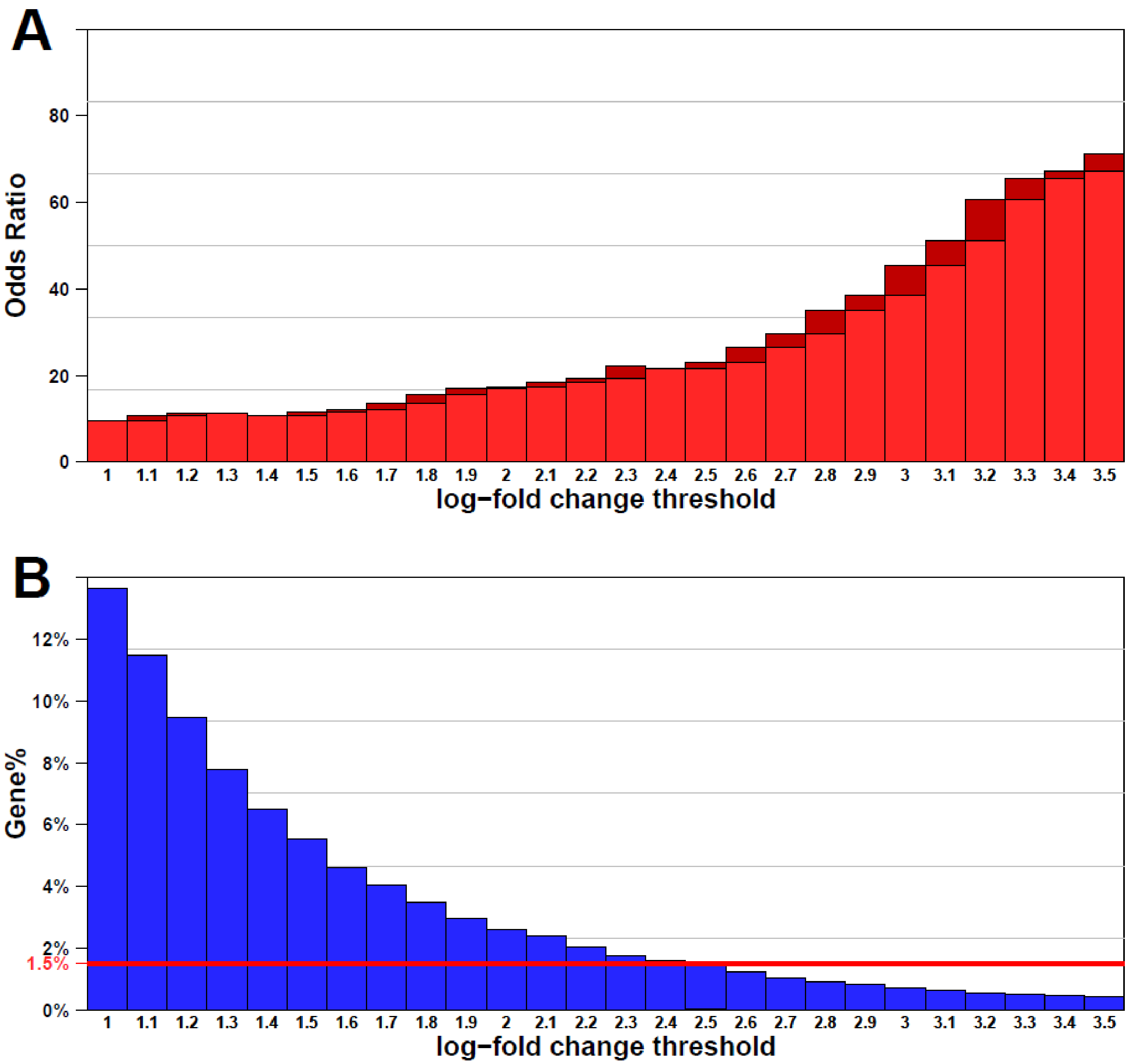

A
80
60
40
20
0
Odds Ratio
1
1.1
1.2
1.3
1.4
1.5
1.6
1.7
1.8
1.9
2
2.1
2.2
2.3
2.4
2.5
2.6
2.7
2.8
2.9
3
3.1
3.2
3.3
3.4
3.5
log−fold change threshold
B
12%
10%
8%
6%
4%
2%
1.5%
0%
Gene%
1
1.1
1.2
1.3
1.4
1.5
1.6
1.7
1.8
1.9
2
2.1
2.2
2.3
2.4
2.5
2.6
2.7
2.8
2.9
3
3.1
3.2
3.3
3.4
3.5
log−fold change threshold

Figure S8

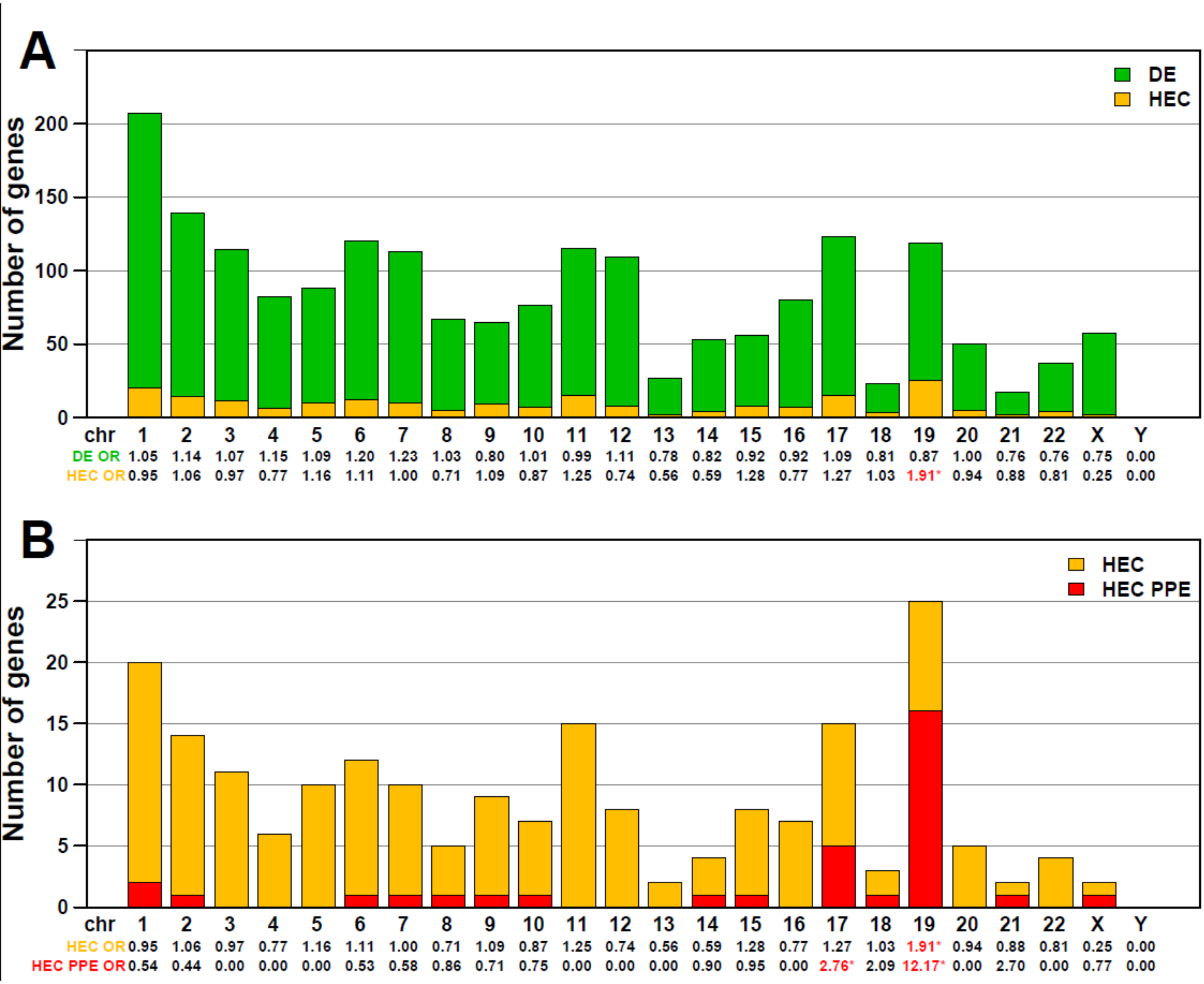
A
Number of genes
0
50
100
150
200
DE
HEC
chr 1 2 3 4 5 6 7 8 9 10 11 12 13 14 15 16 17 18 19 20 21 22 X Y
DE OR 1.05 1.14 1.07 1.15 1.09 1.20 1.23 1.03 0.80 1.01 0.99 1.11 0.78 0.82 0.92 0.92 1.09 0.81 0.87 1.00 0.76 0.76 0.75 0.00
HEC OR 0.95 1.06 0.97 0.77 1.16 1.11 1.00 0.71 1.09 0.87 1.25 0.74 0.56 0.59 1.28 0.77 1.27 1.03 1.91* 0.94 0.88 0.81 0.25 0.00
B
Number of genes
0
5
10
15
20
25
HEC
HEC PPE
chr 1 2 3 4 5 6 7 8 9 10 11 12 13 14 15 16 17 18 19 20 21 22 X Y
HEC OR 0.95 1.06 0.97 0.77 1.16 1.11 1.00 0.71 1.09 0.87 1.25 0.74 0.56 0.59 1.28 0.77 1.27 1.03 1.91* 0.94 0.88 0.81 0.25 0.00
HEC PPE OR 0.54 0.44 0.00 0.00 0.00 0.53 0.58 0.86 0.71 0.75 0.00 0.00 0.00 0.90 0.95 0.00 2.76* 2.09 12.17* 0.00 2.70 0.00 0.77 0.00

Figure S9

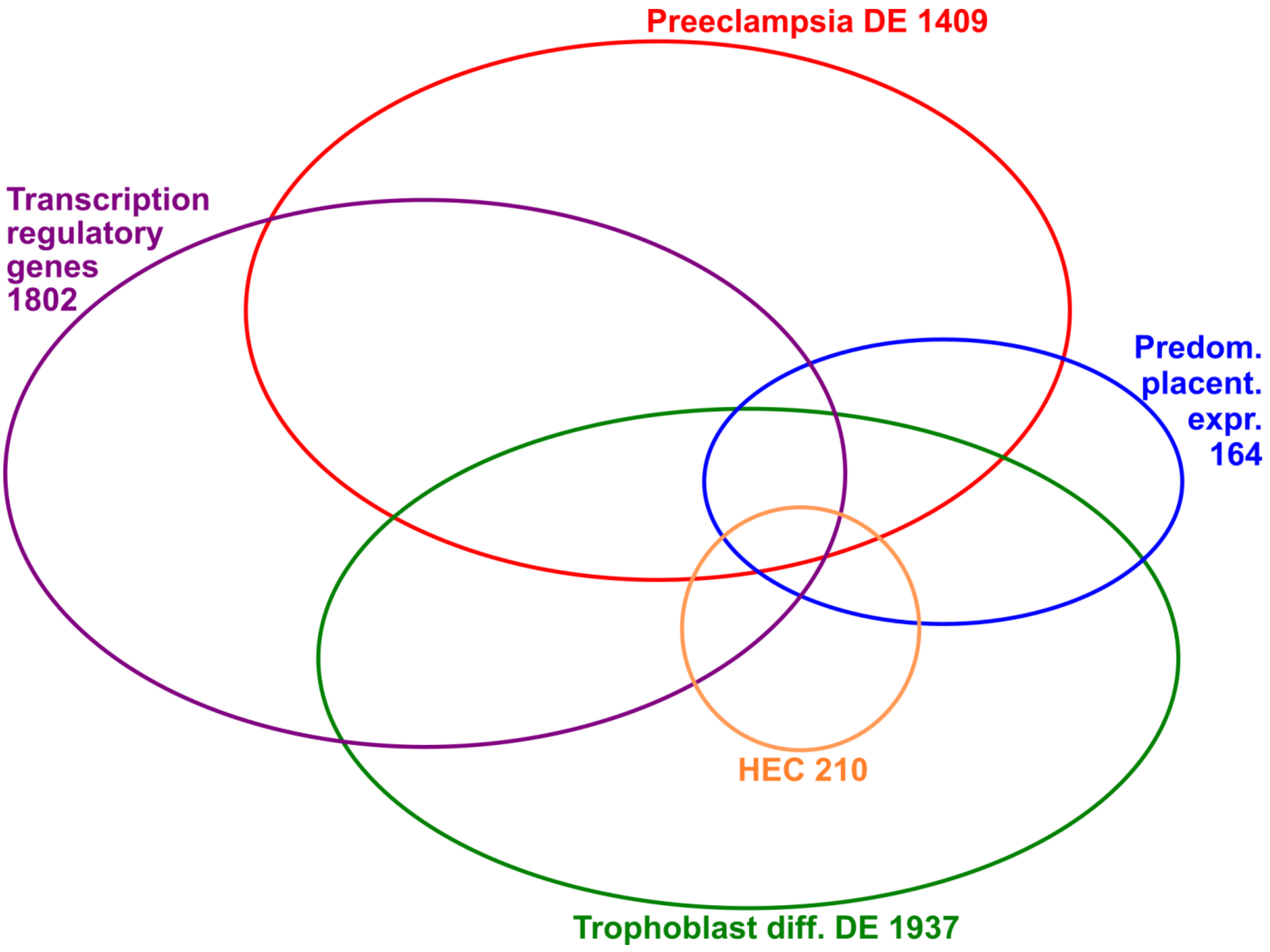
Preeclampsia DE 1409
Transcription regulatory genes 1802
Predom. placent. expr. 164
HEC 210
Trophoblast diff. DE 1937
